# Mitochondrial impairment and melatonin protection in parkinsonian mice do not depend of inducible or neuronal nitric oxide synthases

**DOI:** 10.1371/journal.pone.0183090

**Published:** 2017-08-11

**Authors:** Ana López, Francisco Ortiz, Carolina Doerrier, Carmen Venegas, Marisol Fernández-Ortiz, Paula Aranda, María E. Díaz-Casado, Beatriz Fernández-Gil, Eliana Barriocanal-Casado, Germaine Escames, Luis C. López, Darío Acuña-Castroviejo

**Affiliations:** 1 Instituto de Biotecnología, Centro de Investigación Biomédica, Parque Tecnológico de Ciencias de la Salud, and Departamento de Fisiología, Facultad de Medicina, Universidad de Granada, Granada, Spain; 2 CIBERfes, Ibs.Granada, Complejo Hospitalario de Granada, Granada, Spain; 3 UGC de Laboratorios Clínicos, Complejo Hospitalario de Granada, Granada, Spain; Universidade de Sao Paulo, BRAZIL

## Abstract

MPTP-mouse model constitutes a well-known model of neuroinflammation and mitochondrial failure occurring in Parkinson’s disease (PD). Although it has been extensively reported that nitric oxide (NO^●^) plays a key role in the pathogenesis of PD, the relative roles of nitric oxide synthase isoforms iNOS and nNOS in the nigrostriatal pathway remains, however, unclear. Here, the participation of iNOS/nNOS isoforms in the mitochondrial dysfunction was analyzed in iNOS and nNOS deficient mice. Our results showed that MPTP increased iNOS activity in substantia nigra and striatum, whereas it sharply reduced complex I activity and mitochondrial bioenergetics in all strains. In the presence of MPTP, mice lacking iNOS showed similar restricted mitochondrial function than wild type or mice lacking nNOS. These results suggest that iNOS-dependent elevated nitric oxide, a major pathological hallmark of neuroinflammation in PD, does not contribute to mitochondrial impairment. Therefore, neuroinflammation and mitochondrial dysregulation seem to act in parallel in the MPTP model of PD. Melatonin administration, with well-reported neuroprotective properties, counteracted these effects, preventing from the drastic changes in mitochondrial oxygen consumption, increased NOS activity and prevented reduced locomotor activity induced by MPTP. The protective effects of melatonin on mitochondria are also independent of its anti-inflammatory properties, but both effects are required for an effective anti-parkinsonian activity of the indoleamine as reported in this study.

## Introduction

The participation of factors such as oxidative/nitrosative stress, excitotoxicity, inflammation and mitochondrial dysfunction in the pathogenesis of sporadic Parkinson’s disease (PD) may be studied with the use of 1-methyl-4-phenyl-1,2,3,6-tetrahydropyridine (MPTP) neurotoxin. The active glial metabolite of MPTP, 1-methyl-4-phenylpyridinium (MPP^+^), is taken up into the dopaminergic neurons through the dopamine transporter, and accumulated in the mitochondria of the substantia nigra pars compacta (SNpc) [1–5]. Within the mitochondria, MPP^+^ binds to and inhibits complex I of the electron transport chain (ETC) [6], enhancing the production of superoxide anion (O2^•-^) and other reactive oxygen species (ROS). These ROS produce a sustained oxidative damage to the ETC, subsequent ATP reduction, and nigral cell death [7, 8]. MPP^+^ also induces microglia activation and iNOS expression in the substantia nigra of mice [9], yielding large amounts of NO^•^ that collaborate to neuronal death [10]. Moreover, NO^•^ rapidly reacts with O2^•-^ forming reactive nitrogen species (RNS) including the highly toxic peroxynitrite (ONOO^−^) that irreversibly damage the ETC complexes [11], further promoting neuronal death [12]. Although it is yet unclear why the inflammatory reaction occurs in PD, published data support that the ROS produced in the mitochondria may induce this response [13–21]. Given the role of neuroinflammation in the pathophysiology of PD, the effect of anti-inflammatory drugs in both animal models and epidemiological studies is still controversial [22].

The presence of both constitutive (cNOS) and inducible (iNOS) NOS isoforms in the nigrostriatal tissue has been proposed as responsible of neuronal damage in the striatum (ST) and substantia nigra (SN), respectively, during experimental PD [9]. Previously, the induction of iNOS has been reported in the SN but not in the ST of MPTP-treated mice, whereas the constitutive nNOS enhanced in the latter [23], as well as in other studies iNOS mutant mice were protected against MPTP toxicity [24]. These data further support a role for iNOS/nNOS isoforms in the pathogenesis of PD.

Previous reports showed that the indolamine melatonin (N-acetyl-5-methoxytrytamine, aMT) prevents dopamine auto-oxidation [25] and exerts neuroprotective properties in different types of neurodegeneration, including the MPTP model of PD [26–30]. These effects of melatonin may be related to its antioxidant, anti-inflammatory, and anti-excitotoxic actions. The antioxidant actions involve direct scavenging of both ROS and RNS [31–33], and indirect up-regulation of antioxidant enzymes in multiple tissues including the brain [34, 35]. The anti-inflammatory actions of aMT depend on the inhibition of the expression of iNOS and other Nf-kB-dependent inflammatory mediators [36–39]. A correlation between i-mtNOS expression and mitochondrial failure during inflammation has been also reported in other models [37, 40, 41]. The anti-excitotoxic actions of melatonin are related to the inhibition of the NMDA receptors and the subsequent nNOS activity *in vitro* and in rat striatum *in vivo* [42–44].

A direct relationship between inflammation and mitochondrial damage exists in PD, even before dopaminergic degeneration is observable in this disease. It has been previously described that the induction of iNOS in SN increasing NO^●^ levels in mitochondria after MPTP administration to mice, and also increases nNOS activity and expression in ST, enhancing NO^●^ levels and excitotoxicity [9]. However, whether iNOS/nNOS expression may affect mitochondrial failure in MPTP-induced PD model remains unclear. In this study we used iNOS and nNOS- Knockout mice in order to evaluate the role of these isoforms in mitochondrial failure during sub-acute MPTP model of PD. Furthermore, we proposed that melatonin, which inhibits the expression and activity of iNOS and nNOS as well as improves mitochondrial respiration in other models, could exert neuroprotective effects in these two events independently of the presence/absence of the excess of NO^●^ induced by MPTP.

## Materials and methods

### Animals and treatments

Three months old male mice from four strains were used for this study: iNOS deficient mice (iNOS^-/-^, ref. B6.129P2-Nos2tm1Lau/J) and their respective controls (iNOS^+/+^, ref. C57/Bl/6); and nNOS deficient mice (nNOS^-/-^, ref. B6;129S4-Nos1tm1Plh/J) and their respective wild-type controls (nNOS^+/+^, ref. B6129SF2/J). All strains were obtained from Jackson Laboratory (Sacramento, CA, USA). Mice were housed in clear plastic cages maintained at 22°C ± 1°C and 40% humidity, with a 12 hr light/dark cycle with lights on at 08:00, at the University of Granada’s facility. Mice were feed with tap water and a standard diet ad libitum. All the experiments were performed according to protocols approved by the Institutional Animal Care and Use Committee of the University of Granada (procedures CEEA-2010-273 and 462-CEEA-2013) in accordance with the European Convention for the Protection of Vertebrate Animals used for Experimental and Other Scientific Purposes (CETS # 123) and the Spanish law (R.D. 53/2013). Mice were divided into the following groups: 1- saline-injected control group; 2- MPTP- injected group, and 3- group treated with MPTP and aMT (MPTP+aMT). The doses were administrated in order to study the effects of both treatments in a sub-acute model of PD as previously described [10]. MPTP was dissolved in saline buffer and administered subcutaneously (s.c) in four doses of 15 mg/kg (30 μl/injection) into mice at the first day with 2-h intervals between them. After 24 hr, three additional injections of the same MPTP dose were administrated with the same time interval. Melatonin was dissolved in 30 μL of 2.5% ethanol/saline (v/v) solution and administered at a dose of 10 mg/kg by subcutaneous injection 1 hr before the first dose of MPTP each day. Thirty-two hr after starting treatments, the animals were euthanized using an over-dose of anesthesia (>1.5 mL equithesin/Kg body weight, i.p.) followed by cervical dislocation [23, 45]. Their brains were removed, and fresh SN and ST were dissected for subsequent mitochondria preparation. Fresh isolated mitochondria suspension was used for respiration analysis. Mitochondrial pellets stored at -80°C were used for all other assays.

### Isolation of cytosol and pure mitochondria

Mitochondrial and cytosolic fractions of ST and SN were prepared as described elsewhere [46], with minor modifications. All procedures were carried out at 4°C. Briefly, ST and SN were dissected, weighed, placed into ice-cold buffer (25 mM Tris, 0.5 mM DTT, 10 μg/mL aprotinin, 10 μg/mL leupeptin, 10 μg/mL pepstatin, 1 mM PMSF, pH 7.6) and homogenized (10%, w/v) at 700 rpm in a Teflon pestle. The homogenates were centrifuged at 1,000 g for 3 min. The supernatant was collected in a separate tube and the pellet was resuspended again into ice-cold buffer. The supernatants of these two centrifugations were mixed and centrifuged at 21,200 g for 10 min, yielding the crude mitochondrial and cytosolic fractions. The supernatants of this second centrifugation, which correspond to the crude cytosolic fraction, were frozen at -80°C. The crude mitochondrial pellets were suspended in 15% v/v Percoll prepared in isolation buffer (0.32 M sucrose, 1 mM EDTA-K1, 10 mM Tris-HCl, pH 7.4), in a proportion of 1 g original brain homogenate/10 mL Percoll. Then, this mixture was carefully layered onto a discontinuous Percoll density gradient consisting in 1 mL of 23% (v/v) Percoll layered onto 1 mL of 40% (v/v) Percoll. The samples were centrifuged at 28,200 g for 12 min in an angle-fixed rotor. The bands in the interface between 15% and 23% Percoll layers were aspirated with a syringe, carefully diluted 1:1 with isolation medium, and centrifuged at 16,800 g for 10 min. The pellet was washed in 1 mL isolation buffer to yield a highly pure mitochondrial preparation without contaminating organelles and broken mitochondria [40].

### Determination of melatonin by HPLC

Melatonin in SN and ST was determined by HPLC with fluorescence detection following a method described elsewhere [47]. Frozen mitochondria from SN and ST samples were thawed and sonicated in PBS, pH 7.4, and centrifuged at 3,000 g for 10 min at 4°C. Aliquots of the supernatants were frozen at -80°C for protein determination or mixed (500 μL) with 1 mL chloroform, shaken for 20 min, and centrifuged at 9,000 g for 10 min at 4°C. The organic phase was washed twice with 0.05 m Na_2_CO_3_ buffer, pH 10.25, and 500 μL of the samples was evaporated to dryness in a SPD 2010 SpeedVac System (Fisher Scientific). The residue was then dissolved in 100 μL of mobile phase (100 mM sodium phosphate, 0.1 mM EDTA, and 25% acetonitrile).

Melatonin content was then measured by HPLC (Shimadzu Europe GmbH, Duisburg, Germany) with a 150 × 4.5 mm Waters Sunfire C18 5 μm column (Waters Chromatography, Barcelona, Spain). After stabilizing the column with the mobile phase, samples (20 μL) were injected onto the HPLC system at a 1 mL/min flow rate, and the fluorescence of melatonin was measured in a fluorescence detector (Shimadzu RF-10A XL fluorescence detector) with an excitation and emission wavelength of 285 and 345 nm, respectively. Retention time was 8.9 min. A standard curve for melatonin was constructed with 17.9, 35.9, 71.9, 143.7, and 287.5 pg/mL, and the concentration of melatonin in the samples was calculated according to the peak area. 5-Fluorotryptamine was used as an internal standard [48]. Melatonin levels were expressed in pg/mg protein, measured by Bradford method [49].

### Complex I (NADH:Ubiquinone oxidoreductase) assay

To prepare submitochondrial particles, mitochondrial pellets were frozen and thawed twice, suspended in 160 μL of the incubation medium, and sonicated. Protein concentration in mitochondrial suspension was determined by Bradford method. Complex I activity (NADH CoQ oxidoreductase) was measured in the presence of decylubiquinone and succinate as the rotenone-sensitive decrease in NADH. Briefly, an aliquot of the submitochondrial suspension (0.5 mg protein/mL, final concentration) was added to the reaction mixture containing 0.25 M sucrose, 50 mM KH2PO4, pH 7.4, 1 mM KCN, 10 μg/mL antimycin A, and 50 μM decylubiquinone. After pre-incubation for 3 min at 25°C, the reaction was initiated by the addition of NADH (100 μM, final concentration), and the rate of decrease in the absorbance was monitored at 340 nm for 1.5 min. The incubation in the presence of 1 μM rotenone completely abolished the NADH oxidase activity measured with this method, verifying that this activity corresponds to complex I [50–52]. The activity of the complex I was expressed as nmol of NADH oxidized/min/mgprot.

### Assay of NOS activity

NOS catalyzes the reaction of L-arginine into L-citrulline and NO^●^ in a 1:1 stoichiometric ratio (ie the amount of L-citrulline produced = the amount of NO^●^ produced). As such, measuring L-[^3^H]citrulline derived from radioactively labelled L-[^3^H]arginine can be used to measure NO^●^ production. Therefore, we can state that an increase in NOS activity corresponds to an increase in the production of NO^●^.

Samples from SN and ST were either stored at -80°C for total protein determination by Bradford [49] or used immediately for NOS activity assays, monitoring the conversion of L-[^3^H]arginine to L-[^3^H]citrulline [53]. The final incubation volume was 100 μL and contained 10 μL of sample in 25 mM Tris buffer, 1 mM DTT, 30 μM H4-biopterin, 10 μM FAD, 10 μM L-arginine, 50 nM L-[^3^H]arginine, 0.5 mg/mL BSA, 0.5 mM inosine, and 0.1 mM CaCl_2_ (final concentrations), pH 7.6. The reaction was started by the addition of 10 μL NADPH (0.75 mM final concentration) and continued for 30 min at 37°C. Control incubations were performed in the absence of NADPH. To determine the Ca^2+^-independent iNOS activity, 10 mM EDTA was added to the reaction medium. The reaction was stopped by the addition of 400 fiL cold 0.1 M Hepes containing 10 mM EGTA, 1 mM L-citrulline, pH 5.5. The reaction mixture was decanted into a 2 mL column packed with Dowex-50W ion exchange resin (Na+ form) and eluted with 1.2 mL distilled water. L-[^3^H]citrulline was quantified by liquid scintillation spectroscopy. The retention of L-[^3^H]arginine by the column was greater than 98%. Specific enzyme activity determined by subtracting the control value, which usually amounted less than 1% of the radioactivity added. NOS activity was expressed as pmol of L-[^3^H]citrulline/min/mg protein.

### Quantification of mtDNA damage

Real-time PCR was used to quantify mouse mitochondrial DNA (mtDNA) copy number by relative comparison of glyceraldehyde-3-phosphate dehydrogenase (GAPDH), that is encoded by nuclear DNA (nDNA), to the mitochondrial locus for murine COX I gene, that is transcribed by mitochondrial DNA (mtDNA). Mitochondrial DNA copy number is a critical component of overall mitochondrial health and may provide insight into the processes that either initiate or propagate mitochondrial dysfunction in disease. Primers and detection probe specific to COX I gene were designed by Spinazzola and colleagues using ABI Primer Express software (35) (Fw: 5’-TGCTAGCCGCAGGCATTACT-3’; Rv: 5’-CGGGATCAAAGAAAGTTGTGTTT-3’; mtDNA CO1 MGB-FAM detection probe 5’-TACTACTAACAGACCGCAACC-3’. Primers and detection probe of the gene encoding mouse glyceraldehyde-3-phosphate dehydrogenase were used as nuclear gene standard references, according manufacturer’s instructions. Each PCR reaction was performed in triplicate in a two-step protocol as follows: one cycle at 50°C for 2 min, one cycle at 95°C for 10 min, and then 40 cycles of 95°C for 15 s and 60°C for 1 min. [54]. The values mtDNA levels were normalized by nDNA and the data were expressed in terms of percent relative to wild-type mice.

### Measurement of mitochondrial oxygen consumption

Oxygen consumption was measured by high-resolution respirometry (Oxygraph-2K, OROBOROS Instruments, Innsbruck, Austria) [25]. The oxygraph consists in a two-chamber respirometer with a peltier thermostat and electromagnetic stirrers. The oxygraph was constructed to minimize back-diffusion by using materials that are impermeable for oxygen [27]. The protocol developed to analyse the mitochondrial status by respirometry allowed us to evaluate the effects of MPTP and the involvement of the two isoforms of NOS, constitutive and inducible, in the mitochondrial failure occurring in the primarily affected tissues in Parkinson's disease, SN and ST. Fresh isolated mitochondria from SN and ST were incubated in potassium medium containing 0.5 mM EGTA, 3 mM MgCl2.6H2O, 20 mM taurine, 10 mM KH2PO4, 20 mM HEPES, 200 mM sucrose, and 1g/l BSA, adjusted to pH 7.1 with KOH at 30°C. The medium had previously been equilibrated with air in each chamber completely open (set at 2 mL) at 30°C and stirred at 750 rpm until a stable signal was obtained for calibration at air saturation. A final concentration of 0.2–0.3 mg/mL of protein content in the respiratory buffer was used for the measurements. A protocol was developed for the measurement of the different mitochondrial chain respiratory states, as follows. After closing the chamber, an initial state 2 or routine was measured in the presence of 5 mM malate, glutamate and succinate, substrates for the complexes I and II, which correspond to the basal respiration in absence of ADP. Active respiration (state 3) was then initiating by adding 400 nmol ADP promoting a quick response of the ETC coupled to the oxidative phosphorylation translated into a rapid increase of the oxygen consumption until all ADP has been phosphorylated to ATP. Then oxygen flux falls sharply until oxygen consumption and concentration stabilize in a new plateau called mitochondrial respiratory state 4 or leak state, which corresponds to a non-phosphorylating resting state, higher than state2 because the mitochondria has been activated by the addition of ADP. In order to understand the role of complex I in the mitochondrial failure induced by the neurotoxin after the stabilization of the oxygen flux in state 4, complex I activity was inhibited by the addition of 0.5μM rotenone to the incubation media. The obtained states 2, 3 and leak correspond now to the oxygen consumption through the complex II. The protocol was completed within 20–30 min, time within all the oxygen in the chamber is used, and the data was obtained at 0.2-s intervals using a computer-driven data acquisition system (Datlab, Innsbruck, Austria). The results were expressed as pmol oxygen consumed per minute and per mg of protein at state 2, state 3 or leak state, or as respiratory control index (RCR), i.e., the state 3/Leak state (state 4) respiration ratio. ADP/O ratio was calculated dividing the amount of ADP added by the measured oxygen consumption during state 3 in ng atom oxygen.

### Behavioral analysis

Animals were housed in 4-wall cages with red light during the night and monitored during 4 hr (from 00:00 to 04:00) with a video-tracking system (Smart v. 3.0.03, Panlab Harvard Apparatus, Barcelona, Spain), which allowed the recording of activity and trajectories of mice. The total travelled distance in 4hrs was measured using the Smart video-tracking software. A 100% value was assigned to the distance travelled by control mice and compared to distances travelled by mice treated either with MPTP only or MPTP with aMT.

### Statistical analysis

Eight SN and ST (corresponding to four mice) were mixed and processed together as one sample to obtain a minimal amount of mitochondria for analytical purposes. Thus, the data represent the mean of six experiments (corresponding to 24 mice) performed in triplicate. One-way ANOVA followed by Sidak's multiple comparisons test was used for statistical purposes to compare the effect of the treatments in each tissue separately. Two-way ANOVA followed by Tukey multiple comparisons analysis was used to compare the effect of treatments between the two tissues analyzed, SN and ST. A value of *P* < 0.05 was considered statistically significant.

## Results

### Melatonin concentration increased in mitochondria of SN and ST after its systemic administration

**Fig 1:**
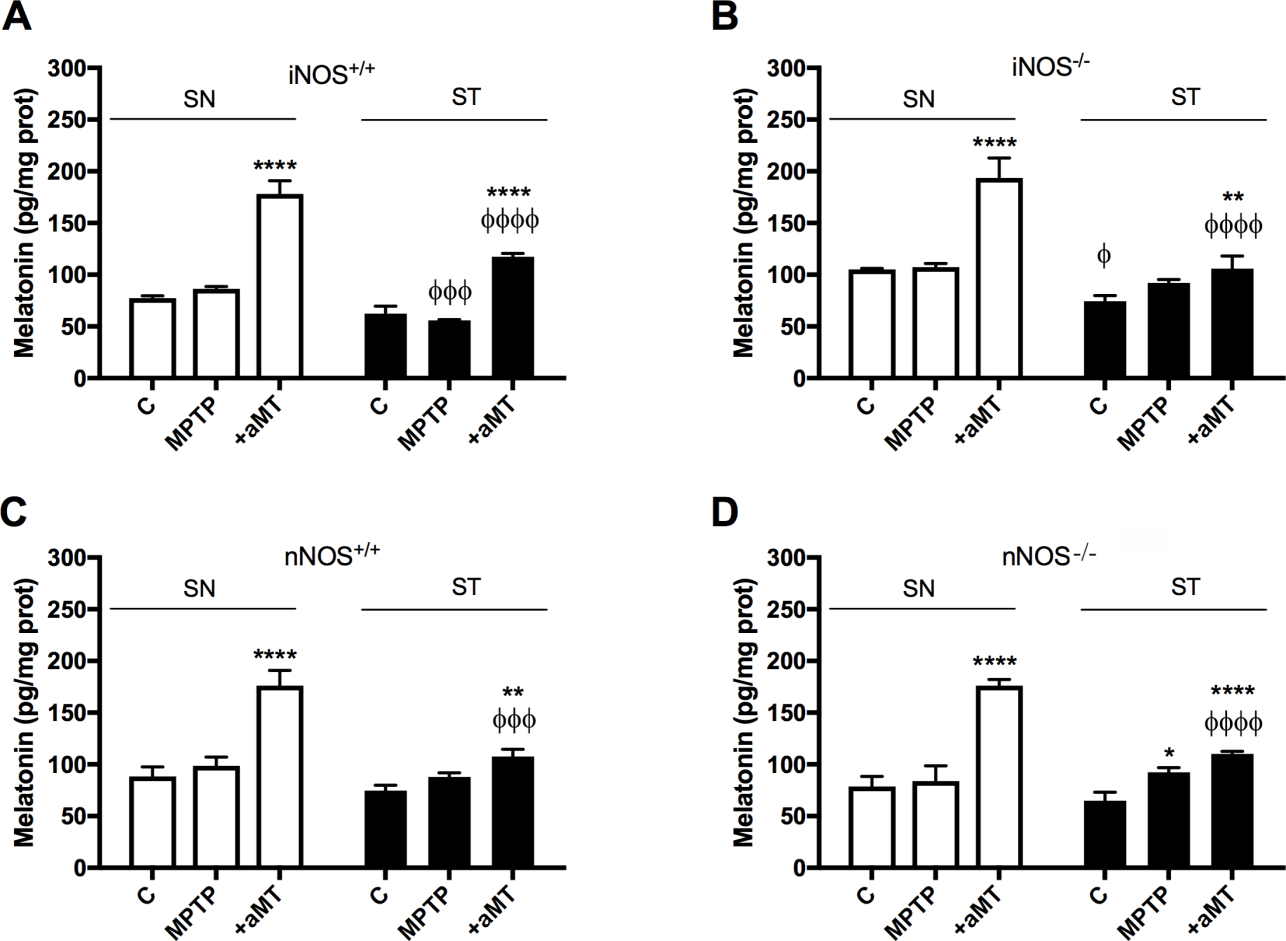
The administration of melatonin increased its levels within the mitochondria of targeted tissues, SN and ST. Graph shows changes in the melatonin levels of mitochondria from SN and ST of iNOS^+/+^ (above left) and iNOS^-/-^ (above right) and SN and ST of nNOS^+/+^ (down left) and nNOS^-/-^ (down right). Mean ± SD of 6 animals per group, triplicated; *P <0.05, **P <0.01, and ****P < 0.0001 *vs*. control; ^Φ^*P* < 0.05, ^ΦΦΦ^*P* < 0.001 and ^ΦΦΦΦ^*P* < 0.0001 *vs*. equivalent group in SN.

To confirm whether melatonin administered to mice reached the SN and ST mitochondria, we measured the melatonin levels in isolated pure mitochondria by HPLC (Fig 1). Within each genotype, the basal content of melatonin in the SN and ST of saline-injected control was not significantly different, except in ST of iNOS^-/-^ mice (P<0.05). MPTP treatment alone increased the mitochondrial melatonin content in the ST of both NOS deficient mice strains, from a mean of 72.83 ± 6.74 pg/mg protein in control group to 88.13 ± 4.34 pg/mg prot after MPTP treatment but this increase was only significant in ST from nNOS^-/-^ mice. After melatonin treatment, the concentration of melatonin within the mitochondria increased significantly in both tissues compared to their respective saline-injected controls in all strains, and this increase was proportionally greater in SN than in ST in all cases. The administration of melatonin increased 2-fold its concentration in the mitochondria from SN of the four mice strains (from a mean of the four control groups of 86.51 ± 10.49 pg/mg protein in control group to 172.49 ± 19.79 pg/mg protein after melatonin treatment, *P* < 0.0001 vs. control). However, the levels of melatonin were proportionally less increased in mitochondria from ST where this increase in melatonin concentration corresponds to a 162 ± 13% of the control level (from a mean of 70.57 ± 4.94 pg/mg protein in control group to 114.92 ± 5.76 pg/mg protein after melatonin treatment).

### MPTP increased iNOS but not nNOS activity in SN and ST of mice, and melatonin treatment prevented this effect

**Fig 2:**
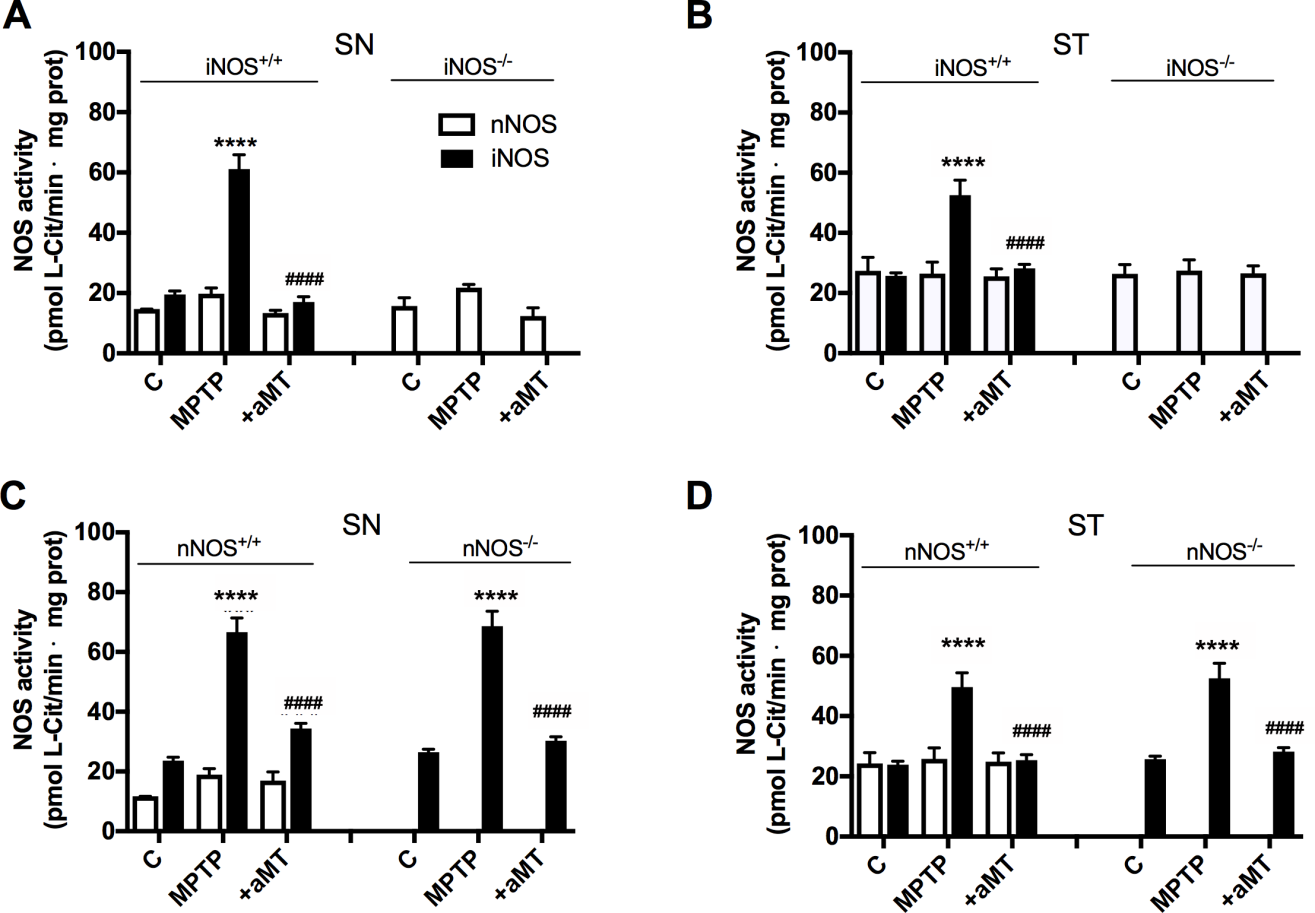
The MPTP treatment caused an increase in the activity of iNOS but not nNOS in SN and ST whereas melatonin treatment restored the activity to control levels. The increase in NOS activity after MPTP administration is abolished in mice lacking iNOS. The graphs show the changes in total NOS and iNOS activities in ST and SN of control mice (left) vs. deficient mice (right) (Mean ± SD of 6 animals per group, triplicated). ****P < 0.0001 *vs*. control; ^####^*P* < 0.0001 *vs*. MPTP.

MPTP administration enhanced iNOS activity in SN (312.63 ± 14.6%; from 19.53 ± 2.1 to 60.93 ± 4.89 pmol of L-[3H]citrulline/min/mg protein) and ST (207.89 ± 12.6%; from 25.21 ± 2.8 to 52.18 ± 5.01 pmol of L-[3H]citrulline/min/mg protein) of iNOS^+/+^ mice (Fig 2A and 2B). MPTP also increased iNOS activity in SN of nNOS^+/+^ and nNOS^-/-^ mice by 324.17% and 292.7% respectively (from 20.15 ± 2.5 to 65.32 ± 5.02 pmol of L-[3H]citrulline/min/mg protein in nNOS^+/+^ and from 24.12 ± 2.47 to 70.6 ± 4.63 pmol of L-[3H]citrulline/min/mg protein in nNOS^-/-^) and in ST in a lesser extent (202.01% in ST from nNOS^+/+^ and 247.78% in ST of nNOS^-/-^; from 24.54 ± 3.02 to 49.59 ± 4.55 pmol of L-[3H]citrulline/min/mg protein and from 20.99 ± 1.87 to 52.01 ± 5.12 pmol of L-[3H]citrulline/min/mg protein, respectively) (Fig 2C and 2D). MPTP, however, did not affect nNOS activity in any mice strain. In both tissues, MPTP increased the total NOS activity due to the inducible component, iNOS, with no changes in nNOS activity, suggesting that the excess of NO^●^ produced in this model of PD depends on enhanced iNOS activity. As expected, mice lacking iNOS or nNOS had not activity of these isoforms, respectively. Our data also show the lack of any compensatory effect for each other’s activity after knocking-out nNOS or iNOS as no increased activity can be seen in either isoform in the SN or ST of saline-injected groups compared to the non-deficient mice. Melatonin administration counteracted the effect of MPTP on iNOS activity, without affecting nNOS, preventing from the production of an excess of NO^●^, and consequently protecting the brain nuclei from nitrosative stress.

### Complex I activity declined in SN and ST after MPTP treatment and melatonin administration counteracted this effect

**Fig 3:**
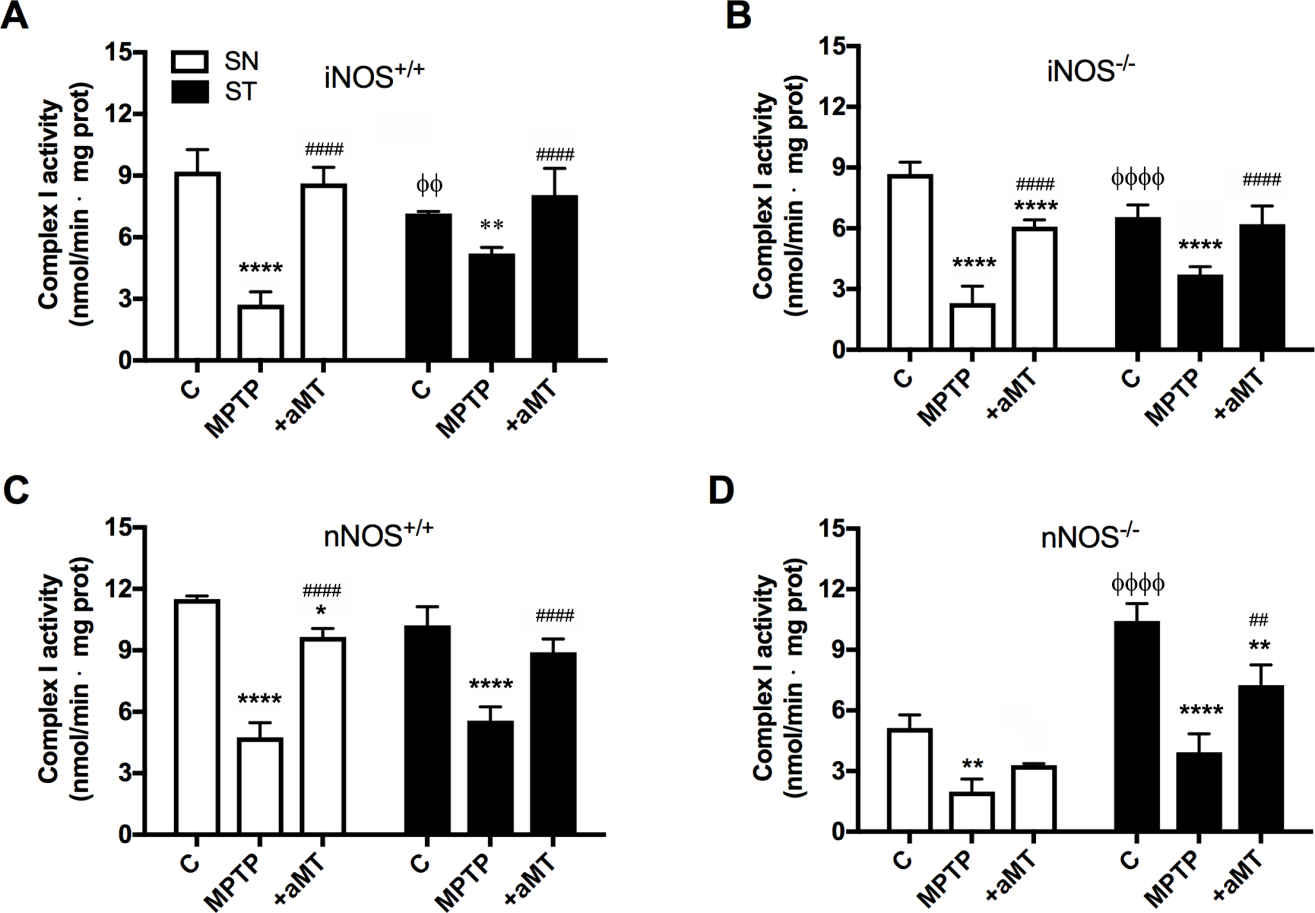
The treatment with MPTP induced a drastic decrease in the activity of the complex I in SN and ST independently of the absence/presence of iNOS/nNOS. The administration of melatonin counteracted this effect. The graphs show the changes in the activity of complex I in SN and ST of iNOS^+/+^ (above left) and iNOS^-/-^ (above right) and SN and ST of nNOS^+/+^ (down left) and nNOS^-/-^ (down right). Mean ± SD of 6 animals per group, triplicated; *P < 0.05, **P < 0.01, and ****P < 0.0001 *vs*. control; *P* < 0.05 ^##^ and ^####^*P* < 0.0001 *vs*. MPTP; ^ΦΦ^*P* < 0.05 and ^ΦΦΦΦ^*P* < 0.0001 *vs*. basal complex I in SN.

Basal activity of complex I was higher in SN than in ST in iNOS^+/+^ and iNOS^-/-^ mice strains (Fig 3A and 3B, *P* < 0.05 and *P* < 0.0001 respectively). Mice lacking nNOS isoform, however, showed higher basal activity of complex I in ST than in SN nuclei (Fig 3D, *P* < 0.0001). In all cases, the administration of MPTP significantly reduced the activity of complex I referred to their respective control value (*P* < 0.0001 for SN of iNOS^+/+^, iNOS^-/-^ and nNOS^+/+^ mice and *P* < 0.01 for SN in nNOS^-/-^). Considering the different basal activity of complex I among the two tissues analyzed in most strains, the MPTP-dependent reduction in this activity was proportionally greater in SN than in ST for all groups except for nNOS^-/-^ mice (Fig 3D). The absence of iNOS did not avoid the decrease in the activity of the complex I after MPTP treatment. Although the absence of nNOS drastically reduced basal complex I activity in SN compared to its wild-type (from 11.5 ± 0.85 nmol/min·mg protein in nNOS^+/+^ to 5.13 ± 1.15 nmol/min·mg protein in nNOS^-/-^), the decay in the percentage of its activity after MPTP treatment was comparable to that in nNOS^+/+^ wild-type mice (61.40% *vs*. 58.53% respectively; from 5.13 ± 1.15 nmol/min·mg protein to 1.98 ± 0.63 nmol/min·mg protein in nNOS^-/-^ and from 11.5 ± 0.85 nmol/min·mg protein to 4.77 ± 0.71 nmol/min·mg protein in nNOS^+/+^). In ST of nNOS^-/-^ mice, however, basal complex I activity was higher than in SN (P>0.0001) and MPTP produced a greater drop in its activity relative to basal value (P>0.0001 in ST vs. P>0.01 in SN). Melatonin administration to MPTP-treated mice recovered complex I activity, an effect independent of the mice strain analyzed; that is, the presence or absence of iNOS/nNOS was unrelated to the melatonin effect.

### MPTP dramatically reduced mitochondrial oxygen consumption, an effect prevented by melatonin administration

**Fig 4:**
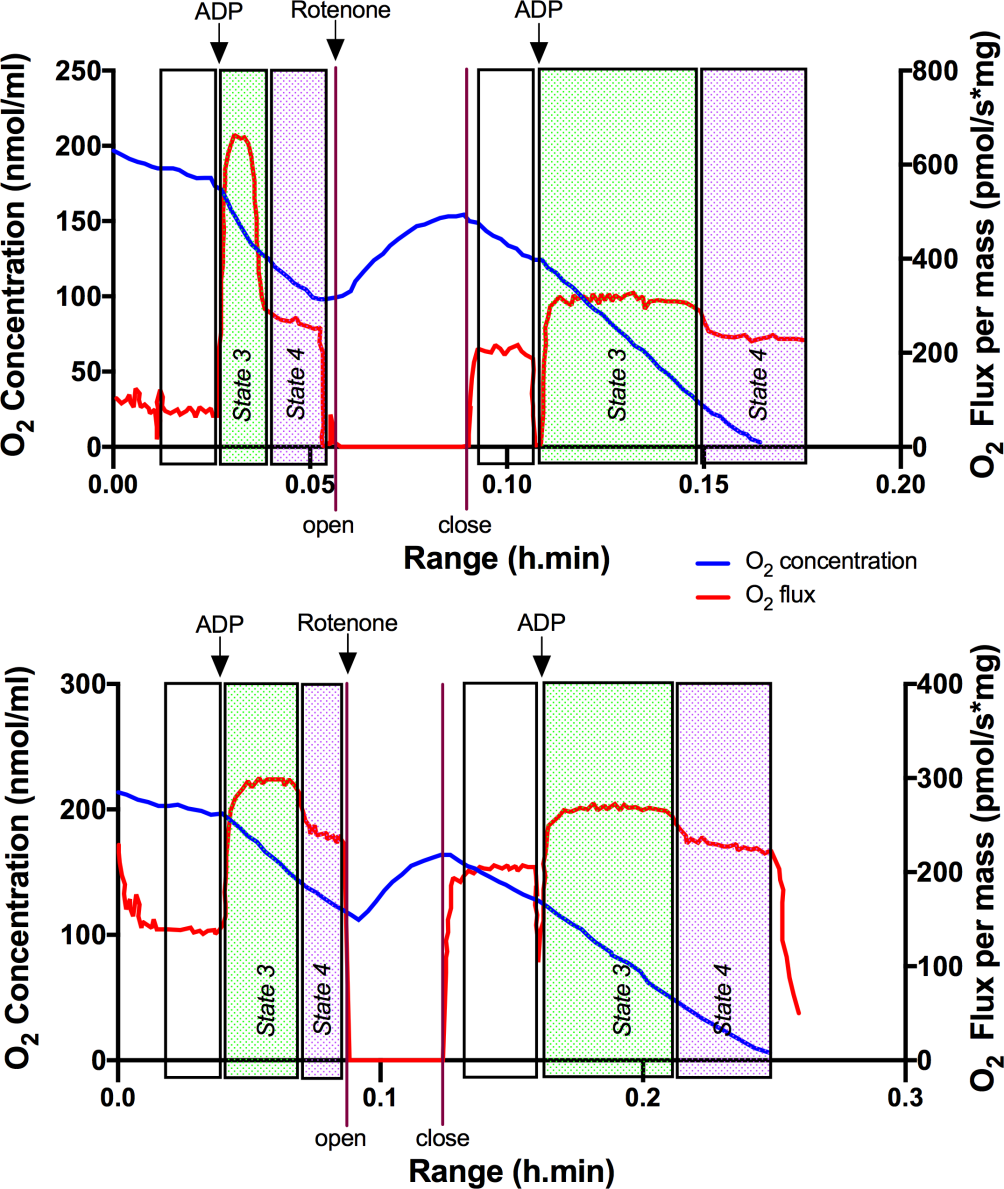
Oxygraph trace of mitochondrial respiration in SN control (above) and MPTP (down) from wild-type for iNOS mice. Oxygen concentration (nmol/mL) and oxygen flux per mass (pmol/s*mg protein) were represented. Black arrows represent activation of respiration by additions of ADP (400 nmol) before and after complex I inhibition by the addition of 2.5 μM of rotenone (white arrow). Changes in state 3 and 4 were then evaluated.

Typical changes in respiratory flux and oxygen concentration of isolated mitochondria from SN of control and MPTP treated iNOS^+/+^ mice are illustrated in Fig 4. Starting from similar values of basal respiration in state 2, MPTP treatment promoted a significant reduction of oxygen consumption after activation of mitochondrial respiration by the addition of ADP. Interestingly, not only the highest value in oxygen flux during state 3 is reduced in SN of MPTP-treated mice, also the time that has been taken for the complete depletion of ADP is much longer, suggesting a slow and less efficient phosphorylation. This effect of MPTP is important and it is not represented in classic state 3-leak state studies. After the inhibition of the complex I by rotenone, the MPTP-dependent decrease on state 3 is prevented, suggesting that the effect of the neurotoxin is targeting specifically the first complex of the ETC but not complex II.

**Fig 5:**
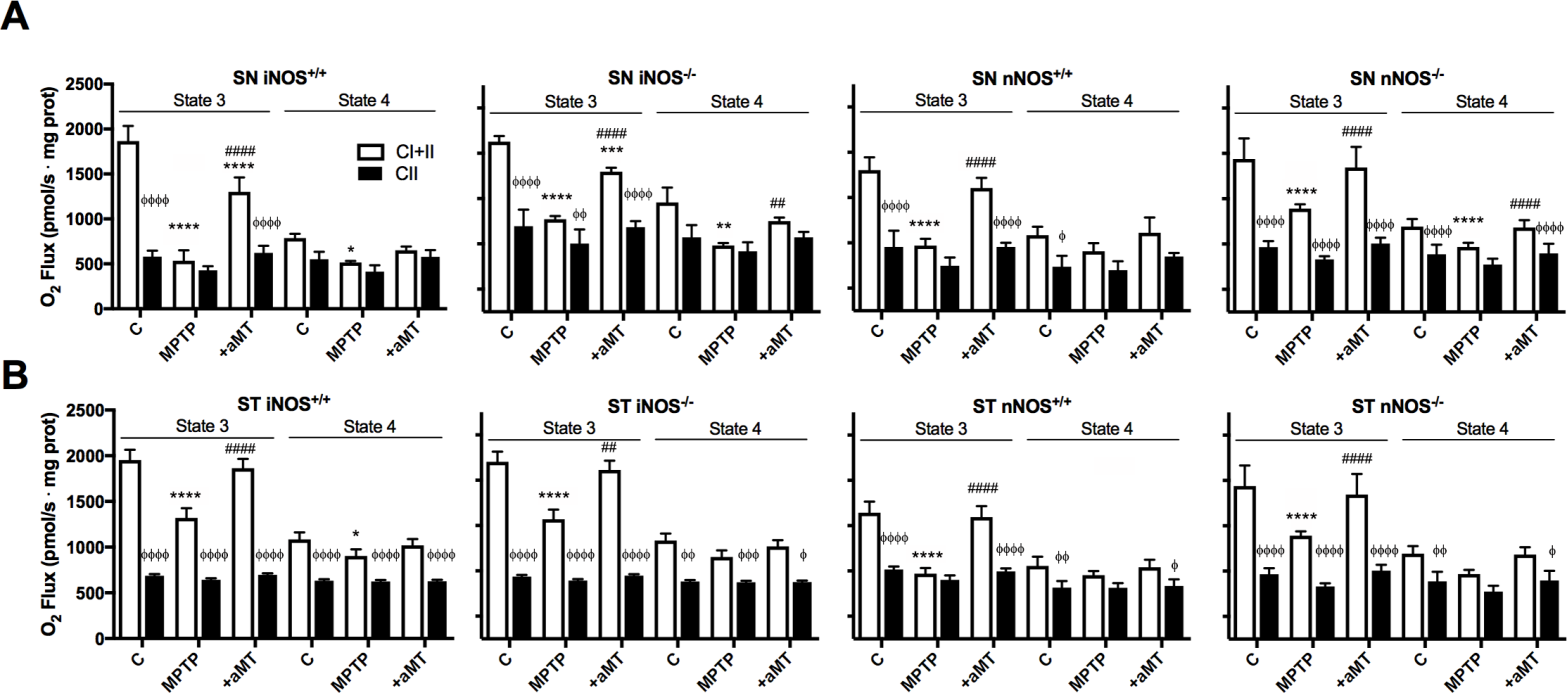
Mitochondrial oxygen consumption decreased sharply after MPTP administration in SN and ST of all strains, an effect prevented by melatonin treatment. More significant changes were found in respiration through complexes I+II. The graphs show the changes in oxygen consumption at state 3 and 4 in SN (A) and ST (B) of iNOS^+/+^, iNOS ^-/-^, nNOS^+/+^ and nNOS ^-/-^ mice respectively, through complexes I and II (dark bars) and through complex II (white bars). Results are shown as the mean ± SD of 4 animals per group, triplicated. *P < 0.*05*, **P < 0.*01*, ***P<0.001 and ****P < 0.*0001 vs*. *saline-injected control;* ^*##*^*P < 0*.*01*, *and* ^*####*^*P < 0*.*0001 MPTP vs*. *MPTP+aMT;* ^Φ^*P* < 0.05, ^ΦΦ^*P* < 0.01, ^ΦΦΦ^*P* < 0.001 and ^ΦΦΦΦ^*P* < 0.0001 CII *vs*. CI+II.

Fig 5 shows the changes in states 3 and leak (state 4) obtained during mitochondrial respiration in SN and ST. The most significant changes occur in MPTP-treated mice during state 3 before the inhibition of complex I by rotenone (CI+CII).This decrease in state 3 was proportionally greater in SN than in ST compared to their respective saline-injected control groups in all strains (mean inhibition of 52.28 ± 6.3% in SN, from 1804 ± 199 pmol/s·mg protein to 938 ± 27 pmol/s·mg protein compared to a 37.71 ± 9.04% inhibition in ST, from a mean of 1832 ± 201 pmol/s·mg protein to 1137 ± 262 pmol/s·mg protein after MPTP treatment) (Fig 5A). Compared to state 3, state 4 was less affected by MPTP or by rotenone inhibition, confirming that the ETC dysfunction after MPTP is due to damaged complex I. Interestingly, MPTP treatment resulted in a smaller decrease of the state 3 referred to its saline-injected control in the SN from iNOS^-/-^ than in SN from iNOS^+/+^ (45.56% vs. 71.33% of reduction respectively; from 1878.42 ± 62.58 pmol/s·mg protein to 1022.51 ± 33.8 pmol/s·mg protein in iNOS^-/-^ and from 1865.20 ± 169.39 pmol/s·mg protein to 534.75 ± 157.3 pmol/s·mg protein in iNOS^+/+^). Similar changes were found in the SN of nNOS^-/-^ and nNOS^+/+^ (44.89% vs. 53.84% of reduction respectively; from 1374 ± 126.34 pmol/s·mg protein to 757.13 ± 59.01 pmol/s·mg protein in nNOS^-/-^ and from 1552.29 ± 142.49 pmol/s·mg protein to 720.63 ± 135 pmol/s·mg protein in nNOS^+/+^). However, mice lacking nNOS showed a lesser reduction of the active state after MPTP in the ST than nNOS^+/+^ mice (32.52% vs. 48.32% respectively; from 1689.32 ± 227 pmol/s·mg protein to 1139.95 ± 48.21 pmol/s·mg protein in nNOS^-/-^ and from 1393.32 ± 122 pmol/s·mg protein to 719.95 ± 62.24 pmol/s·mg protein in nNOS^+/+^). Importantly, although depletion of iNOS does not prevent the reduction in the state 3, absence of iNOS seems to increase both resting (state 4) and active (state 3) respiration, effect restricted to mitochondria from the SN, suggesting a bigger role of this isoform in this tissue where iNOS activity is more sharply increased. Melatonin treatment counteracted the harmful effects of MPTP, restoring significantly the state 3 respiration in mitochondria from SN and ST of all animal strains, and with minimal effects on leak state.

The respiratory control ratio (RCR), the ratio of state 3/Leak state, is the best indicator of well-coupled isolated mitochondria. MPTP significantly reduced RCR ratio measured during CI+II respiration in all cases, except in SN of iNOS^-/-^ and ST of nNOS^-/-^ (Table 1). The effects of MPTP on RCR ratio calculated during CII respiration showed minimal effects and significant changes were only found in nNOS^+/+^ mice.

**Table 1:** Control respiratory index (RCR) in ST and SN mitochondria.

	Substantia nigra						Striatum					
	CI+CII			CII			CI+CII			CII		
Group	Control	MPTP	+aMT	Control	MPTP	+aMT	Control	MPTP	+aMT	Control	MPTP	+aMT
iNOS^+/+^	2.37 ± 0.05	1.04 ± 0.05****	2.00 ± 0.09^####^^,^ ****	1.06 ± 0.01	1.04 ± 0.01	1.08 ± 0.01	1.80 ± 0.07	1.46 ± 0.05***	1.83 ± 0.08^####^	1.09 ± 0.05	1.03 ± 0.04	1.11 ± 0.02
iNOS^-/-^	1.56 ± 0.1	1.40 ± 0.08	1.54 ± 0.09^#^	1.15 ± 0.11	1.13 ± 0.04	1.14 ± 0.07	1.88 ± 0.11	1.40 ± 0.09***	1.87 ± 0.08^####^	1.37 ± 0.08	1.22 ± 0.11	1.30 ± 0.07
nNOS^+/+^	1.86 ± 0.09	1.10 ± 0.06****	1.57 ± 0.09^###^^,^ ***	1.45 ± 0.05	1.11 ± 0.08****	1.18 ± 0.09****	1.73 ± 0.08	1.02 ± 0.06****	1.70 ± 0.09^####^	1.36 ± 0.04	1.16 ± 0.05**	1.28 ± 0.08
nNOS^-/-^	1.58 ± 0.1	1.30 ± 0.08***	1.60 ± 0.05^####^	1.04 ± 0.03	1.02 ± 0.02	1.05 ± 0.04	1.79 ± 0.12	1.59 ± 0.11	1.71 ± 0.10^#^	1.12 ± 0.09	1.11 ± 0.09	1.16 ± 0.08

Data are shown as the mean ± SEM.

**p < 0.01

***p<0.001 *and*

****p < 0.0001 *vs*. control

^###^*p* < 0.001 and

^####^*p* < 0.0001 vs. MPTP.

The efficiency of oxidative phosphorylation can be measured as the ratio of nmoles of ADP consumed per nanoatoms of oxygen consumed during ADP-stimulated respiration (ADP/O ratio). Interestingly, we found that MPTP had a greater effect in ST than in SN reducing significantly ADP/O through CI+II in ST of all strains and in SN from nNOS^-/-^ mice. The significantly decreased respiratory control ratio (RCR) found in SN from wild-type mice, iNOS^+/+^ and nNOS^+/+^, did not result in a lesser oxidative phosphorylation efficiency in this tissue. Again, MPTP had lower effects on the ADP/O ratio during CII respiration (Table 2).

**Table 2:** Values of the ADP:O ratio in ST and SN mitochondria.

	Substantia nigra							Striatum					
	CI+CII			CII				CI+CII			CII		
Group	Control	MPTP	+aMT	Control	MPTP		+aMT	Control	MPTP	+aMT	Control	MPTP	+aMT
iNOS^+/+^	2.49 ± 0.21	2.01 ± 0.09	2.25 ± 0.38	1.34 ± 0.04		1.14 ± 0.05*	1.31 ± 0.06^#^	3.08 ± 0.15	2.43 ± 0.17****	2.83 ± 0.2^#^	1.58 ± 0.19	1.26 ± 0.08**	1.32 ± 0.05**
iNOS^-/-^	2.51 ± 0.2	2.62 ± 0.37	2.36 ± 0.39	1.10 ± 0.05		1.05 ± 0.15	1.13 ± 0.11	3.14 ± 0.08	2.33 ± 0.03****	3.05 ± 0.02^####^	1.49 ± 0.04	1.28 ± 0.13	1.37 ± 0.16
nNOS^+/+^	3.17 ± 0.17	2.70 ± 0.31	2.88 ± 0.17	1.28 ± 0.11		1.20 ± 0.12	1.27 ± 0.1	2.66 ± 0.12	2.10 ± 0.14****	2.26 ± 0.05***	1.55 ± 0.08	1.36 ± 0.09	1.57 ± 0.04
	2.46 ± 0.1	1.78 ± 0.18*	2.23 ± 0.38^##^	1.51 ± 0.04		1.07 ± 0.09****	1.39 ± 0.02^###^	2.61 ± 0.06	1.36 ± 0.18****	2.16 ± 0.13^####^^,^ ****	1.17 ± 0.06	1.08 ± 0.02	1.57 ± 0.002^####^^,^ ****

Data are shown as the mean ± SEM.

*p < 0.05

**p < 0.01

***p < 0.001 and

****p < 0.0001 *vs*. control

^#^*p* < 0.05

^##^*p* < 0.01

^###^*p* < 0.001 and

^####^*p* < 0.0001 vs. MPTP.

In analysing the ability of melatonin to rescue the effect of MPTP, two comparisons were made. Firstly, whether the MPTP+aMT treatment was significantly different from the MPTP only treatment, where a significant increase would show melatonin ameliorating the toxic effect of MPTP. And secondly, whether the MPTP+aMT treatment was significantly different from the control group, where non-significance would show melatonin completely rescuing these parameters to control levels.

In all cases where MTPT significantly decreases RCR and ADP/O ratio (ie. all except for the RCR in SN of nNOS^+/+^ through CII and ADP/O in ST of iNOS^+/+^ through CII), melatonin increases significantly these values, and in many occasions, to control levels. Therefore, melatonin treatment rescues the coupling between the electron transport chain and the oxidative phosphorylation as well as the efficiency of oxidative phosphorylation.

### NOS isoforms and melatonin treatment have different effects on mtDNA content

**Fig 6:**
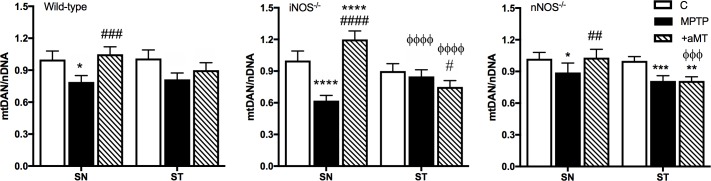
Content of mtDNA was minimally affected by the presence/absence of NOS isoforms and melatonin. The graph shows the mtDNA/nDNA ratio in ST and SN control (A), nNOS-/- (B) and iNOS-/- (C) mice treated with MPTP and melatonin. Mean ± SD of 6 animals per group, triplicated; *P < 0.05, **P < 0.01 ***P < 0.001 and ****P < 0.0001 *vs*. saline-injected control; ^*#*^*P < 0*.*05*, ^*##*^*P < 0*.*01*, ^*###*^*P < 0*.*001 and* ^*####*^*P < 0*.*0001 MPTP vs*. *MPTP+aMT;* ^ΦΦΦ^*P* < 0.001 and ^ΦΦΦΦ^*P* < 0.0001 *vs*. equivalent group in SN.

To evaluate the role of the presence/absence of constitutive and inducible NOS isoforms and melatonin treatment on mtDNA in our MPTP model, mtDNA expression was measured by RT-PCR. The ratio mtDNA/nDNA was used as an indicator of mitochondrial genomic damage and/or biogenesis of the organelle (Fig 6). MPTP treatment induced a reduction in the ratio mtDNA/nDNA compared to their saline-injected controls in all strains and in both ST and SN, except in ST from iNOS^-/-^ mice. Interestingly, the lack of iNOS could not prevent the mitochondrial DNA depletion induced by MPTP in SN, where the most significant change was found compared to the saline-injected control group (P<0.0001 in iNOS^-/-^ vs. P<0.05 in SN from wild-type and nNOS^-/-^). Melatonin administration showed a very different effect in ST and SN of all mice strains. In ST, the effect of the indolamine was minimal and unable to rescue the MPTP-dependent reduced mtDNA/nDNA ratio in each mouse strain. In contrast, in SN, melatonin treatment promoted an increase in mitochondrial DNA levels reestablishing control values, including SN from iNOS^-/-^ where the most drastic changes were found compared to saline-injected control group.

### Melatonin prevented the bradykinesia induced by MPTP

**Fig 7:**
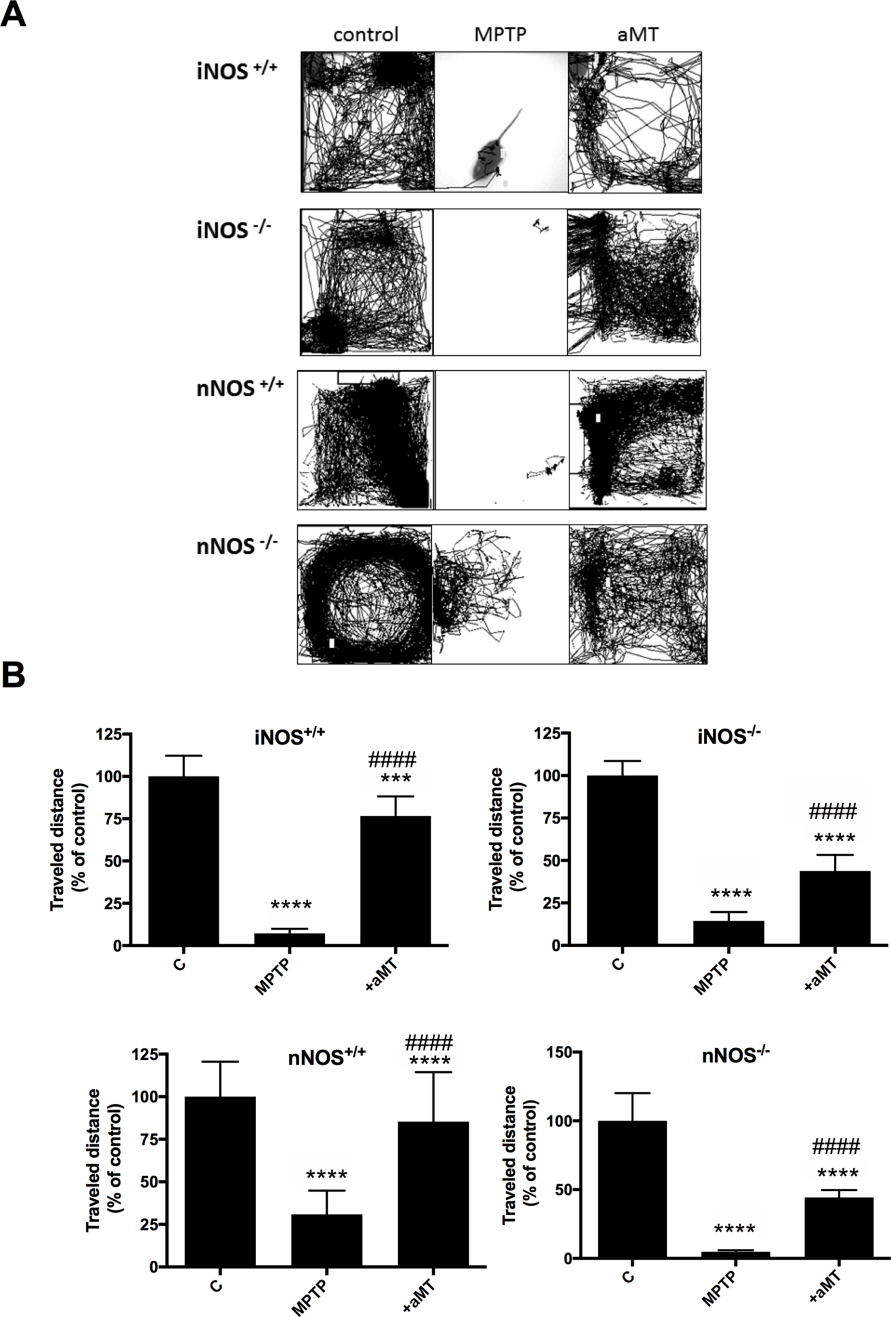
MPTP induced dramatic changes in mice locomotor activity, which were prevented by melatonin. Graphs show the changes in trajectories (A) and travelled distance (B) of iNOS^+/+^, iNOS ^-/-^, nNOS^+/+^ and nNOS ^-/-^ mice respectively, during 4 hr at night, in control, MPTP and aMT treated groups. Mean ± SD of 8 animals per group. ***P <0.001 and ****P <0.0001 *vs*. saline-injected control; ^####^*P* < 0.0001 *vs*. MPTP treatment.

Mice were housed in 4-wall cages with red light during the night and the motion was monitored during 4 hr from 00:00 to 04:00 am. The results of the behavioral analysis based on the video tracking recording of travelled distance and trajectories are shown in Figs 3A and 7B. MPTP administration sharply reduced the normal locomotor activity of wild-type and mutant mice. nNOS^-/-^ mice were affected by MPTP in a lesser extent that the other three mice strains as shown in Fig 7A. Mice lacking nNOS showed an additional 18% loss of motion vs. nNOS^+/+^ after MPTP administration referred to their respective saline-injected control. However, the MPTP-induced decrease found in iNOS^-/-^ mice occurs in a lesser extent compared to iNOS^+/+^. Melatonin treatment significantly rescued the MPTP-dependent reduction in travelled distance (P<0.0001 in all cases) and maintained almost normal locomotor activity in the wild-type mice strains. A typical recording video of the locomotor activity of a mouse from each strain is shown in the attached video (S1 Movie).

## Discussion

This report shows by the first time that the mitochondrial respiration impairment in the SN and ST of MPTP-treated mice is independent of the iNOS induction in these tissues in a sub-acute model of PD. Our results show that MPTP treatment increased the production of NO^●^ derived from increased activity of iNOS and hence unrelated to nNOS activity in SN and ST. In spite of this specific iNOS-dependent production of NO^●^, depletion of iNOS or nNOS, failed to rescue the drastic decrease in oxygen consumption induced by MPTP in all strains. This dysfunction is reflected in lower values of state 3 and consequently, RCR and ADP/O. Oxygen flux during state 3 was more affected in SN than in ST after MPTP treatment. However, SN and ST showed a different scenario. Mice lacking iNOS showed a lesser reduction in all respirometric parameters in the SN compared to iNOS^+/+^, suggesting a potential role of the isoform in this tissue, where significantly higher levels of NO^●^ are produced. On the other hand, absence of nNOS resulted in smaller changes in oxygen flux in ST, although RCR and ADP/O were equally affected compared to nNOS^+/+^ mice. This could indicate that ST is more sensitive to changes in oxygen flux than SN. These data suggest a different role of nNOS and iNOS in mitochondrial respiration failure in SN and ST. Thus, the parkinsonism-like effects of MPTP on mitochondrial respiration here described probably depend on other effects of the neurotoxin. These MPTP-dependent effects could include the selective inhibition of complex I and subsequent respiratory deficits and oxidative stress, rather than a consequence of iNOS/nNOS induction.

Our results show that MPTP administration enhanced iNOS activity in SN and ST of wild-type mice, without changes in nNOS. These findings suggest a direct role of iNOS, and not nNOS, in the well-reported overproduction of NO^●^ associated with typical parkinsonian inflammation [9, 55]. Elevated iNOS activity yields to an excess of NO^●^ that induces dopaminergic cell death by nitrosative/oxidizing damage and further respiratory deficiency [12, 23, 56].

The active form of the neurotoxin MPTP, MPP^+^, selectively inhibits mitochondrial complex I, enhancing ROS generation by this respiratory complex [57, 58]. Administration of MPTP in this model reduced complex I activity by 66% in SN and 26% in ST. However, this reduction was comparable in wild-type and mice lacking iNOS or nNOS revealing that the absence of iNOS does not influence the activity of the complex I in MPTP-treated mice. This finding may be explained by a sustained NO^●^-independent inhibition of complex I by MPP^+^. Data from septic mice showed that the inhibition of mitochondrial complex I activity was related to the excess of NO^●^ produced by iNOS due to the inflammatory process, and this inhibition disappeared in iNOS-deficient mice [40, 59]. A different scenario appears in MPTP-treated mice where complex I remains inhibited in mice lacking iNOS.

Complex I together with complex III are the main loci involved in ROS production in the mitochondria [60]. It has been previously shown that MPP^+^ increases the formation of ROS within the mitochondria, leading to oxidative damage, reduced respiratory function, and dysregulation of mitochondrial dynamics and homeostasis [61–63]. Additionally, the SN contains high levels of iron and it is highly sensitive to ROS which in turn, induce mitochondrial failure and dopaminergic cell death [64]. In contrast, our respiration data revealed that mitochondrial respiratory deficit occurs upon MPTP treatment regardless the presence/absence of iNOS and the consequent excess of NO^●^. MPTP reduced the state 3 of mitochondrial respiration in all strains, especially in SN. The time elapsed for the complete depletion of ADP was also longer in the presence of MPTP, suggesting a slow and less efficient phosphorylation in parkinsonian mice compared to saline-injected mice. The severe reduction of mitochondrial respiration was also observed after acute and chronic administration of MPTP in other mouse models [65, 66]. Decreased state 3 values after MPTP treatment resulted in a lower RCR and ADP/O ratios than in control groups, especially notorious when both complexes I and II are active. The effects of lacking iNOS in mitochondrial respiration may reflect some side effect of NO^●^ in mitochondrial inner membrane such as protein nitration [67, 68], causing a minimal reduction in RCR ratio in iNOS^-/-^ mice. Further studies in chronic models of PD may yield information whether a sustained effect of NO^●^ results in a major bioenergetic impairment.

It has been previously demonstrated that melatonin improves mitochondrial efficiency, harnessing the activity of the four respiratory complexes, and reducing ROS generation in healthy mitochondria [69]. Melatonin administration also counteracts the inhibition of complex I induced by MPTP [26, 70], recovering the production of ATP and the cellular survival. Here, we found that administered melatonin accumulates in the mitochondria of SN and ST, and rescues decreased complex I activity induced by MPTP in all mouse strains used. Our data revealed that the absence of iNOS does not influence the activity of the complex I in MPTP mice.

Melatonin also lowers the expression of iNOS in MPTP-treated mice and cell models, reducing the inflammatory response to the neurotoxin [23, 71]. Although we observed a decrease in the activity of iNOS after melatonin treatment, the irrelevance of presence/absence of this isoform in respiration failure suggests that complex I might be one of the first targets of melatonin. Therefore, the recovery of mitochondrial complex I seems to involve a direct mechanism that is independent on its anti-inflammatory properties. We thus asked whether melatonin could support oxidative phosphorylation in brain mitochondria, and, if so, whether it may rescue mitochondrial respiration depressed by MPP^+^-mediated complex I blockade. Consistent with the effects on complex I, melatonin restored state 3, RCR, and ADP/O ratios, in many occasions to control values, in both SN and ST of all mice strains treated with MPTP, improving mitochondrial function. These effects also reflect the role of melatonin preventing the loss of the mitochondrial membrane potential that may trigger the permeability transition pore [72]. Recently published, the beneficial effect of melatonin counteracting mitochondrial impairment induced by MPTP in respiration was also shown in a zebrafish model of Parkinson’s disease. MPTP treated zebrafish embryos showed a reduced mitochondrial complex I activity and diminished expression of mitochondrial degradation-related genes parkin/PINK1/DJ-1, all leading to mitochondrial dysfunction. Interestingly, removal of MPTP treatment did not slow down the progression of the disease and fish developed severe motor defects. Melatonin, administered together with MPTP or once MPTP treatment was stopped, prevented and recovered normal mitochondrial function and gene expression [28].

The MPTP treatment also induced a clear decrease in the mtDNA/nDNA ratio. The impairment of mtDNA homeostasis plays an important role in the pathogenesis of neuronal loss in parkinsonian patients and methods to assess the copy number of mtDNA have been suggested to be used as biomarker for the etiology of PD [73]. However, the mechanism by which mtDNA copy-number loss participates in brain aging and neurodegeneration remains unclear. The mtDNA copy number increases with age in healthy individuals in spite of accumulating deletions. In the substantia nigra of patients with PD, the mtDNA homeostasis is impaired and mtDNA copy number does not increase resulting in depletion of the wild-type mtDNA population [74]. Substantia nigra is shown to be especially vulnerable to mtDNA copy-number impairment and inherited mtDNA-depletion disorders [75]. The reduction in the mtDNA/nDNA ratio we observed may contribute to the respiratory deficiency found in our model, thus participating in the cascade of neurodegeneration [74]. The absence of iNOS or nNOS could not prevent the mitochondrial DNA depletion induced by MPTP. The effects of melatonin were however restricted to the SN, restoring the control values in all mice strains, further supporting that melatonin positively affects mitochondria function reducing their oxidative damage. Moreover, interaction of melatonin with cytochrome c [76] and binding with high affinity to complex I [77, 78], also collaborates in explaining the specific effects of the indoleamine on mitochondrial function. Together, our data demonstrate that melatonin treatment is able to maintain proper mitochondrial respiration function, protecting from the impairment of the oxidative phosphorylation system caused by MPTP treatment.

In addition, the neuroprotective impact of melatonin treatment here reported was confirmed by behavioral improvement. The length of trajectories in MPTP-injected mice was significantly shorter than saline-injected controls in the open field. Melatonin treated mice performed better than the MPTP-parkinsonian mice (see video). The ameliorated locomotor deficit induced by melatonin treatment has been also shown in chronic MPTP mouse model of PD [79]. Of note, the attenuation of MPTP-induced motor loss by melatonin was smaller in knockout than in wild-type mice but significantly increased with respect to MPTP-only treated mice. These results suggest that mice lacking iNOS are less affected to the loss of motion promoted by MPTP administration.

In summary, MPTP increases the production of the inflammation marker NO^●^ by increasing the activity of the iNOS isoform. Moreover, our data also exclude the participation of nNOS on mitochondrial respiration failure in MPTP-treated mice, because the presence or absence of nNOS did not modify mitochondrial respiration status either in SN or ST. In addition, the inhibition of mitochondrial complex I by MPTP seems to be independent of iNOS activation and leads to a deep mitochondrial respiration dysfunction in all mouse strains. On the other hand, melatonin blunts the MPTP toxicity and recovers mitochondria oxygen consumption and locomotor activity.

**Fig 8:**
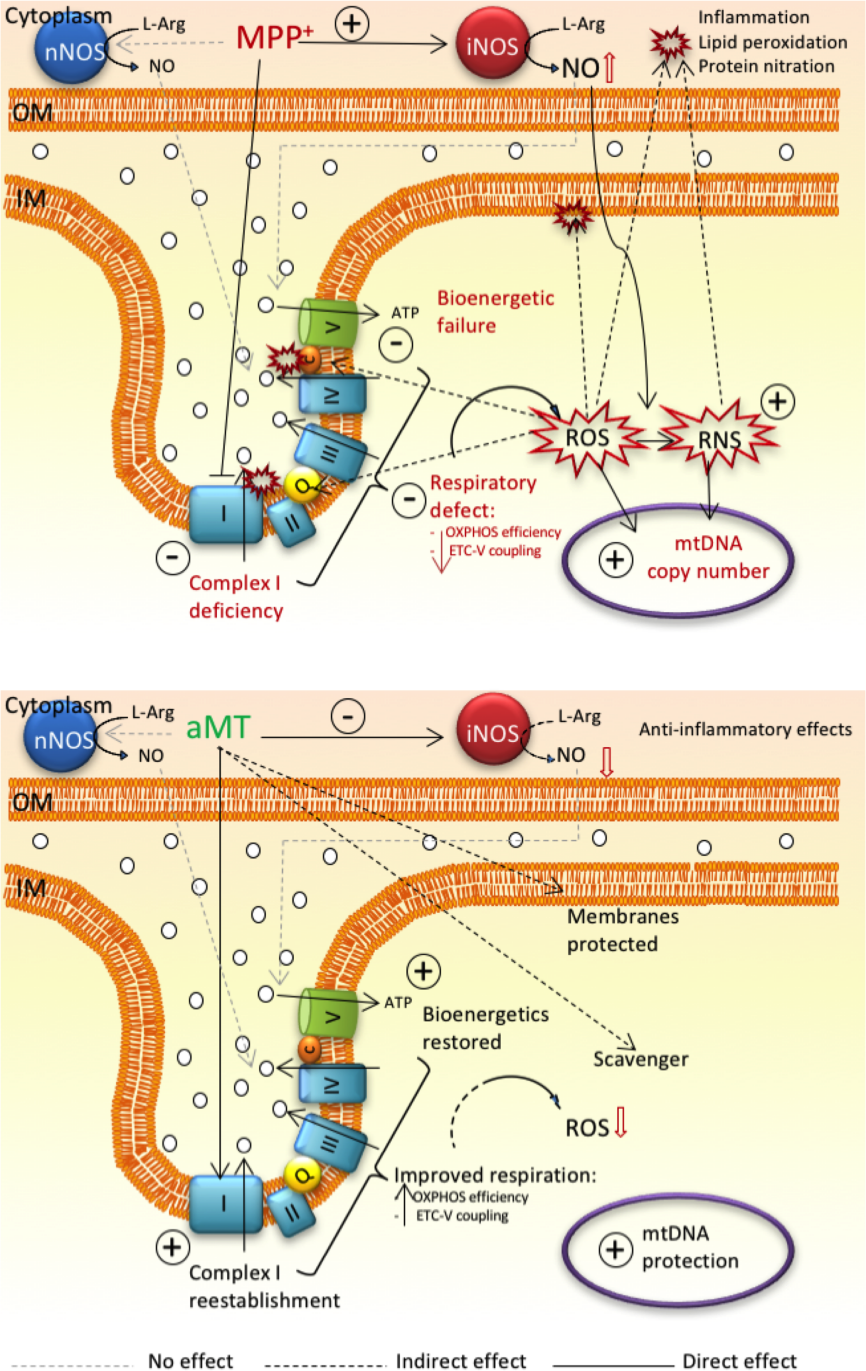
Scheme summarizing the main findings reported in this study. The glial metabolite of MPTP, MPP+, enters into dopaminergic neurons in the SN and accumulates in the mitochondria. where it specifically binds to and inhibits complex I of the ETC. This leads to mitochondrial respiration failure, reducing ETC-OXPHOS coupling as well as the efficiency of the oxidative phosphorylation. MPP+ also promotes an increase in the production of NO^●^ by activation of the inducible isoform iNOS, whereas nNOS activity remains unchanged. Depletion of either iNOS or nNOS did not prevent the decrease in oxygen consumption, suggesting that MPTP-induced respiratory defects and consequent ROS production run independently of iNOS activation. The interplay of these two MPTP-dependent consequences, respiration failure and NO^●^ production, could generate a positive feedback loop where inflammation and oxidative damage are prevalent and cause neurodegeneration. This misbalance generates an excess of ROS by electron leak that, in combination with RNS, is known to induce the expression of inflammatory cytokines and membrane oxidative damage. Melatonin that also accumulates in the mitochondria, harnesses the activity of the respiratory complexes including the inhibition of complex I induced by MPTP as a first action. Melatonin administration restored the coupling between the ETC and OXPHOS, rescued mitochondrial respiration and reduced ROS production, by reducing electron leak and directly scavenging free radicals. On the other hand, the indoleamine also inhibits iNOS activation, reducing NO^●^ production and consequently reducing inflammation that closes the cycle.

Together, the data indicate that mitochondrial respiration impairment and iNOS-induced high levels of NO^●^ as a neuroinflammation marker, are running in parallel promoting neurodegeneration in the MPTP model of PD. The sub-acute MPTP-model of PD constitutes a scientific approach that allows to analyze the inflammatory reaction and the impairment in mitochondrial oxygen consumption separately. This pathophysiological duality explains why anti-inflammatory drugs are not effective against PD, because they may blunt inflammation but not mitochondrial respiration deficits. Melatonin becomes a type of drug that meets this double approach; the indoleamine recovers mitochondrial bioenergetics and blocks neuroinflammation. The accumulation of melatonin in the mitochondria after its administration may favor its beneficial effects (Fig 8). More efficient strategies against PD should target simultaneously mitochondria and inflammation, and melatonin itselft may serve as template to design new therapeutic drugs.

## Supporting information

S1 Movie Typical recording video of the locomotor activity of a mouse from each strain monitored during 4 hr (from 00:00 to 04:00) with Smart video-tracking system, which allowed the recording of activity and trajectories of mice. (MPG)

S1 File Supplementary tables showing the statistical analyses for all data presented. (DOCX)

## References

[1] Przedborski S, Tieu K, Perier C, Vila M. MPTP as a mitochondrial neurotoxic model of Parkinson's disease. JBioenergBiomembr. 2004;36(4):375–9. 10.1023/B:JOBB.0000041771.66775.d5 15377875

[2] Burns RS. Subclinical damage to the nigrostriatal dopamine system by MPTP as a model of preclinical Parkinson's disease: a review. Acta neurologica Scandinavica Supplementum. 1991;136:29–36. 1801534 10.1111/j.1600-0404.1991.tb05017.x

[3] Buneeva OA, Medvedev AE. [Mitochondrial disfunction in Parkinson's disease]. Biomeditsinskaia khimiia. 2011;57(3):246–81. 21863741 10.18097/pbmc20115703246

[4] Maret G, Testa B, Jenner P, el Tayar N, Carrupt PA. The MPTP story: MAO activates tetrahydropyridine derivatives to toxins causing parkinsonism. Drug metabolism reviews. 1990;22(4):291–332. doi: 10.3109/03602539009041087 2253555 10.3109/03602539009041087

[5] Watanabe Y, Himeda T, Araki T. Mechanisms of MPTP toxicity and their implications for therapy of Parkinson's disease. Medical science monitor: international medical journal of experimental and clinical research. 2005;11(1):RA17–23. 15614202

[6] Greenamyre JT, Sherer TB, Betarbet R, Panov AV. Complex I and Parkinson's disease. IUBMBLife. 2001;52(3–5):135–41. 10.1080/15216540152845939 11798025

[7] Przedborski S, Ischiropoulos H. Reactive oxygen and nitrogen species: weapons of neuronal destruction in models of Parkinson's disease. AntioxidRedoxSignal. 2005;7(5–6):685–93. 10.1089/ars.2005.7.685 15890013

[8] Tretter L, Sipos I, Adam-Vizi V. Initiation of neuronal damage by complex I deficiency and oxidative stress in Parkinson's disease. NeurochemRes. 2004;29(3):569–77. 10.1023/b:nere.0000014827.94562.4b 15038604

[9] Liberatore GT, Jackson-Lewis V, Vukosavic S, Mandir AS, Vila M, McAuliffe WG, et al Inducible nitric oxide synthase stimulates dopaminergic neurodegeneration in the MPTP model of Parkinson disease. NatMed. 1999;5(12):1403–9. 10.1038/70978 10581083

[10] Brown TP, Rumsby PC, Capleton AC, Rushton L, Levy LS. Pesticides and Parkinson's disease—is there a link? EnvironHealth Perspect. 2006;114(2):156–64. 10.1289/ehp.8095 PMC1367825 16451848

[11] Brown GC, Borutaite V. Nitric oxide inhibition of mitochondrial respiration and its role in cell death. Free RadicBiolMed. 2002;33(11):1440–50. 10.1016/s0891-5849(02)01112-7 12446201

[12] Zhang L, Dawson VL, Dawson TM. Role of nitric oxide in Parkinson's disease. PharmacolTher. 2006;109(1–2):33–41. 10.1016/j.pharmthera.2005.05.007 16005074

[13] Zhou RB, Yazdi AS, Menu P, Tschopp J. A role for mitochondria in NLRP3 inflammasome activation. Nature. 2011;469(7329):221–5. doi: 10.1038/nature09663 21124315 10.1038/nature09663

[14] Shukla V, Mishra SK, Pant HC. Oxidative stress in neurodegeneration. Advances in pharmacological sciences. 2011;2011:572634 doi: 10.1155/2011/572634 21941533 10.1155/2011/572634 PMC3177364

[15] Reynolds A, Laurie C, Mosley RL, Gendelman HE. Oxidative stress and the pathogenesis of neurodegenerative disorders. International review of neurobiology. 2007;82:297–325. doi: 10.1016/S0074-7742(07)82016-2 17678968 10.1016/S0074-7742(07)82016-2

[16] Halliwell B, Jenner P. Impaired clearance of oxidised proteins in neurodegenerative diseases. Lancet. 1998;351(9114):1510. 10.1016/S0140-6736(05)78898-X 9605824

[17] Noack H, Possel H, Rethfeldt C, Keilhoff G, Wolf G. Peroxynitrite mediated damage and lowered superoxide tolerance in primary cortical glial cultures after induction of the inducible isoform of NOS. Glia. 1999;28(1):13–24. 10498818 10.1002/(sici)1098-1136(199910)28:1<13::aid-glia2>3.0.co;2-l

[18] Gao HM, Liu B, Zhang W, Hong JS. Synergistic dopaminergic neurotoxicity of MPTP and inflammogen lipopolysaccharide: relevance to the etiology of Parkinson's disease. FASEB journal: official publication of the Federation of American Societies for Experimental Biology. 2003;17(13):1957–9. 12923073 10.1096/fj.03-0203fje

[19] Ebadi M, Sharma SK. Peroxynitrite and mitochondrial dysfunction in the pathogenesis of Parkinson's disease. Antioxidants & redox signaling. 2003;5(3):319–35. 12880486 10.1089/152308603322110896

[20] Goralski KB, Renton KW. Brain inflammation enhances 1-methyl-4-phenylpyridinium-evoked neurotoxicity in rats. Toxicology and applied pharmacology. 2004;196(3):381–9. doi: 10.1016/j.taap.2003.12.022 15094308 10.1016/j.taap.2003.12.022

[21] Shin CY, Choi JW, Jang ES, Ju C, Kim WK, Kim HC, et al Dehydroepiandrosterone inhibits the death of immunostimulated rat C6 glioma cells deprived of glucose. Brain research. 2001;922(2):267–75. 11743959 10.1016/s0006-8993(01)03185-7

[22] Bassani TB, Vital MA, Rauh LK. Neuroinflammation in the pathophysiology of Parkinson's disease and therapeutic evidence of anti-inflammatory drugs. Arquivos de neuro-psiquiatria. 2015;73(7):616–23. doi: 10.1590/0004-282X20150057 26200058 10.1590/0004-282X20150057

[23] Tapias V, Escames G, Lopez LC, Lopez A, Camacho E, Carrion MD, et al Melatonin and its brain metabolite N(1)-acetyl-5-methoxykynuramine prevent mitochondrial nitric oxide synthase induction in parkinsonian mice. JNeurosciRes. 2009;87(13):3002–10. 10.1002/jnr.22123 19437546

[24] Dehmer T, Lindenau J, Haid S, Dichgans J, Schulz JB. Deficiency of inducible nitric oxide synthase protects against MPTP toxicity in vivo. JNeurochem. 2000;74(5):2213–6. 10800968 10.1046/j.1471-4159.2000.0742213.x

[25] Khaldy H, Escames G, Leon J, Vives F, Luna JD, Acuna-Castroviejo D. Comparative effects of melatonin, L-deprenyl, Trolox and ascorbate in the suppression of hydroxyl radical formation during dopamine autoxidation in vitro. JPineal Res. 2000;29(2):100–7. 10981823 10.1034/j.1600-079x.2000.290206.x

[26] Khaldy H, Escames G, Leon J, Bikjdaouene L, Acuna-Castroviejo D. Synergistic effects of melatonin and deprenyl against MPTP-induced mitochondrial damage and DA depletion. NeurobiolAging. 2003;24(3):491–500. 10.1016/s0197-4580(02)00133-1 12600724

[27] Naskar A, Prabhakar V, Singh R, Dutta D, Mohanakumar KP. Melatonin enhances L-DOPA therapeutic effects, helps to reduce its dose, and protects dopaminergic neurons in 1-methyl-4-phenyl-1,2,3,6-tetrahydropyridine-induced Parkinsonism in mice. Journal of pineal research. 2015;58(3):262–74. doi: 10.1111/jpi.12212 25626558 10.1111/jpi.12212

[28] Diaz-Casado ME, Lima E, Garcia JA, Doerrier C, Aranda P, Sayed RK, et al Melatonin rescues zebrafish embryos from the parkinsonian phenotype restoring the parkin/PINK1/DJ-1/MUL1 network. Journal of pineal research. 2016;61(1):96–107. doi: 10.1111/jpi.12332 27064726 10.1111/jpi.12332

[29] Su LY, Li H, Lv L, Feng YM, Li GD, Luo R, et al Melatonin attenuates MPTP-induced neurotoxicity via preventing CDK5-mediated autophagy and SNCA/alpha-synuclein aggregation. Autophagy. 2015;11(10):1745–59. doi: 10.1080/15548627.2015.1082020 26292069 10.1080/15548627.2015.1082020 PMC4824603

[30] Tan DX, Manchester LC, Qin L, Reiter RJ. Melatonin: A Mitochondrial Targeting Molecule Involving Mitochondrial Protection and Dynamics. International journal of molecular sciences. 2016;17(12). 10.3390/ijms17122124 PMC5187924 27999288

[31] Tan DX, Manchester LC, Reiter RJ, Plummer BF, Limson J, Weintraub ST, et al Melatonin directly scavenges hydrogen peroxide: a potentially new metabolic pathway of melatonin biotransformation. Free RadicBiolMed. 2000;29(11):1177–85. 10.1016/s0891-5849(00)00435-4 11121726

[32] Acuna-Castroviejo D, Martin M, Macias M, Escames G, Leon J, Khaldy H, et al Melatonin, mitochondria, and cellular bioenergetics. JPineal Res. 2001;30(2):65–74. 11270481 10.1034/j.1600-079x.2001.300201.x

[33] Reiter RJ, Tan DX, Manchester LC, Qi W. Biochemical reactivity of melatonin with reactive oxygen and nitrogen species: a review of the evidence. Cell biochemistry and biophysics. 2001;34(2):237–56. doi: 10.1385/CBB:34:2:237 11898866 10.1385/CBB:34:2:237

[34] Antolin I, Rodriguez C, Sainz RM, Mayo JC, Uria H, Kotler ML, et al Neurohormone melatonin prevents cell damage: effect on gene expression for antioxidant enzymes. FASEB J. 1996;10(8):882–90. 8666165 10.1096/fasebj.10.8.8666165

[35] Carretero M, Escames G, Lopez LC, Venegas C, Dayoub JC, Garcia L, et al Long-term melatonin administration protects brain mitochondria from aging. Journal of pineal research. 2009;47(2):192–200. doi: 10.1111/j.1600-079X.2009.00700.x 19573039 10.1111/j.1600-079X.2009.00700.x

[36] Crespo E, Macias M, Pozo D, Escames G, Martin M, Vives F, et al Melatonin inhibits expression of the inducible NO synthase II in liver and lung and prevents endotoxemia in lipopolysaccharide-induced multiple organ dysfunction syndrome in rats. FASEB J. 1999;13(12):1537–46. 10463945

[37] Escames G, Leon J, Macias M, Khaldy H, Acuna-Castroviejo D. Melatonin counteracts lipopolysaccharide-induced expression and activity of mitochondrial nitric oxide synthase in rats. FASEB J. 2003;17(8):932–4. doi: 10.1096/fj.02-0692fje 12670878 10.1096/fj.02-0692fje

[38] Garcia JA, Volt H, Venegas C, Doerrier C, Escames G, Lopez LC, et al Disruption of the NF-kappaB/NLRP3 connection by melatonin requires retinoid-related orphan receptor-alpha and blocks the septic response in mice. FASEB journal: official publication of the Federation of American Societies for Experimental Biology. 2015. 10.1096/fj.15-273656 26045547

[39] Ortiz F, Acuna-Castroviejo D, Doerrier C, Dayoub JC, Lopez LC, Venegas C, et al Melatonin blunts the mitochondrial/NLRP3 connection and protects against radiation-induced oral mucositis. Journal of pineal research. 2015;58(1):34–49. doi: 10.1111/jpi.12191 25388914 10.1111/jpi.12191

[40] Lopez LC, Escames G, Tapias V, Utrilla P, Leon J, Acuna-Castroviejo D. Identification of an inducible nitric oxide synthase in diaphragm mitochondria from septic mice: its relation with mitochondrial dysfunction and prevention by melatonin. IntJBiochemCell Biol. 2006;38(2):267–78. 10.1016/j.biocel.2005.09.008 16223598

[41] Ortiz F, Garcia JA, Acuna-Castroviejo D, Doerrier C, Lopez A, Venegas C, et al The beneficial effects of melatonin against heart mitochondrial impairment during sepsis: inhibition of iNOS and preservation of nNOS. Journal of pineal research. 2014;56(1):71–81. doi: 10.1111/jpi.12099 24117944 10.1111/jpi.12099

[42] Escames G, Leon J, Lopez LC, Acuna-Castroviejo D. Mechanisms of N-methyl-D-aspartate receptor inhibition by melatonin in the rat striatum. JNeuroendocrinol. 2004;16(11):929–35. 15584934 10.1111/j.1365-2826.2004.01250.x

[43] Leon J, Macias M, Escames G, Camacho E, Khaldy H, Martin M, et al Structure-related inhibition of calmodulin-dependent neuronal nitric-oxide synthase activity by melatonin and synthetic kynurenines. Molecular pharmacology. 2000;58(5):967–75. 11040043 10.1124/mol.58.5.967

[44] Leon J, Escames G, Rodriguez MI, Lopez LC, Tapias V, Entrena A, et al Inhibition of neuronal nitric oxide synthase activity by N1-acetyl-5-methoxykynuramine, a brain metabolite of melatonin. JNeurochem. 2006;98(6):2023–33. 16945113 10.1111/j.1471-4159.2006.04029.x

[45] Acuna-Castroviejo D, Tapias V, Lopez LC, Doerrier C, Camacho E, Carrion MD, et al Protective effects of synthetic kynurenines on 1-methyl-4-phenyl-1,2,3,6-tetrahydropyridine-induced parkinsonism in mice. Brain research bulletin. 2011;85(3–4):133–40. doi: 10.1016/j.brainresbull.2011.03.008 21419832 10.1016/j.brainresbull.2011.03.008

[46] Rice JC, Lindsay JG. Subcellular fractionation. In: Graham JM, editor. Rickwood1997.

[47] Venegas C, Garcia JA, Escames G, Ortiz F, Lopez A, Doerrier C, et al Extrapineal melatonin: analysis of its subcellular distribution and daily fluctuations. Journal of pineal research. 2012;52(2):217–27. doi: 10.1111/j.1600-079X.2011.00931.x 21884551 10.1111/j.1600-079X.2011.00931.x

[48] Sastre Torano J, Rijn-Bikker P, Merkus P, Guchelaar HJ. Quantitative determination of melatonin in human plasma and cerebrospinal fluid with high-performance liquid chromatography and fluorescence detection. Biomedical chromatography: BMC. 2000;14(5):306–10. doi: 10.1002/1099-0801(200008)14:5<306::AID-BMC986>3.0.CO;2-7 10960829 10.1002/1099-0801(200008)14:5<306::AID-BMC986>3.0.CO;2-7

[49] Bradford MM. A rapid and sensitive method for the quantitation of microgram quantities of protein utilizing the principle of protein-dye binding. AnalBiochem. 1976;72:248–54. 10.1016/0003-2697(76)90527-3 942051

[50] Barrientos A. In vivo and in organello assessment of OXPHOS activities. Methods. 2002;26(4):307–16. doi: 10.1016/S1046-2023(02)00036-1 12054921 10.1016/S1046-2023(02)00036-1

[51] Barrientos A, Fontanesi F, Diaz F. Evaluation of the mitochondrial respiratory chain and oxidative phosphorylation system using polarography and spectrophotometric enzyme assays. Current protocols in human genetics. 2009;Chapter 19:Unit19 3. 10.1002/0471142905.hg1903s63 PMC2771113 19806590

[52] Lopez-Fabuel I, Resch-Beusher M, Carabias-Carrasco M, Almeida A, Bolanos JP. Mitochondrial Complex I Activity is Conditioned by Supercomplex I-III2-IV Assembly in Brain Cells: Relevance for Parkinson's Disease. Neurochemical research. 2017. 10.1007/s11064-017-2191-2 28197854

[53] Bredt DS, Snyder SH. Nitric oxide mediates glutamate-linked enhancement of cGMP levels in the cerebellum. ProcNatlAcadSciUSA. 1989;86(22):9030–3. 10.1073/pnas.86.22.9030 PMC298426 2573074

[54] Spinazzola A, Viscomi C, Fernandez-Vizarra E, Carrara F, D'Adamo P, Calvo S, et al MPV17 encodes an inner mitochondrial membrane protein and is mutated in infantile hepatic mitochondrial DNA depletion. NatGenet. 2006;38(5):570–5. 10.1038/ng1765 16582910

[55] Terzioglu M, Galter D. Parkinson's disease: genetic versus toxin-induced rodent models. The FEBS journal. 2008;275(7):1384–91. doi: 10.1111/j.1742-4658.2008.06302.x 18279376 10.1111/j.1742-4658.2008.06302.x

[56] Tsang AH, Chung KK. Oxidative and nitrosative stress in Parkinson's disease. Biochimica et biophysica acta. 2009;1792(7):643–50. doi: 10.1016/j.bbadis.2008.12.006 19162179 10.1016/j.bbadis.2008.12.006

[57] Cleeter MW, Cooper JM, Schapira AH. Irreversible inhibition of mitochondrial complex I by 1-methyl-4-phenylpyridinium: evidence for free radical involvement. Journal of neurochemistry. 1992;58(2):786–9. 1729421 10.1111/j.1471-4159.1992.tb09789.x

[58] Jenner P. Oxidative mechanisms in nigral cell death in Parkinson's disease. Movement disorders: official journal of the Movement Disorder Society. 1998;13 Suppl 1:24–34. 9613715

[59] Escames G, Lopez LC, Tapias V, Utrilla P, Reiter RJ, Hitos AB, et al Melatonin counteracts inducible mitochondrial nitric oxide synthase-dependent mitochondrial dysfunction in skeletal muscle of septic mice. JPineal Res. 2006;40(1):71–8. 16313501 10.1111/j.1600-079X.2005.00281.x

[60] Liu Y, Fiskum G, Schubert D. Generation of reactive oxygen species by the mitochondrial electron transport chain. JNeurochem. 2002;80(5):780–7. 11948241 10.1046/j.0022-3042.2002.00744.x

[61] Bueler H. Impaired mitochondrial dynamics and function in the pathogenesis of Parkinson's disease. ExpNeurol. 2009;218(2):235–46. 10.1016/j.expneurol.2009.03.006 19303005

[62] Chen H, Chan DC. Mitochondrial dynamics—fusion, fission, movement, and mitophagy—in neurodegenerative diseases. HumMolGenet. 2009;18(R2):R169–R76. 10.1093/hmg/ddp326 PMC2758711 19808793

[63] Drechsel DA, Patel M. Role of reactive oxygen species in the neurotoxicity of environmental agents implicated in Parkinson's disease. Free RadicBiolMed. 2008;44(11):1873–86. 10.1016/j.freeradbiomed.2008.02.008 PMC2723777 18342017

[64] Speciale SG. MPTP: insights into parkinsonian neurodegeneration. NeurotoxicolTeratol. 2002;24(5):607–20. 10.1016/s0892-0362(02)00222-2 12200192

[65] Tieu K, Ischiropoulos H, Przedborski S. Nitric oxide and reactive oxygen species in Parkinson's disease. IUBMBLife. 2003;55(6):329–35. 10.1080/1521654032000114320 12938735

[66] Patki G, Che Y, Lau YS. Mitochondrial dysfunction in the striatum of aged chronic mouse model of Parkinson's disease. Frontiers in aging neuroscience. 2009;1:3 doi: 10.3389/neuro.24.003.2009 20552054 10.3389/neuro.24.003.2009 PMC2874410

[67] Murray J, Taylor SW, Zhang B, Ghosh SS, Capaldi RA. Oxidative damage to mitochondrial complex I due to peroxynitrite: identification of reactive tyrosines by mass spectrometry. The Journal of biological chemistry. 2003;278(39):37223–30. doi: 10.1074/jbc.M305694200 12857734 10.1074/jbc.M305694200

[68] Aulak KS, Miyagi M, Yan L, West KA, Massillon D, Crabb JW, et al Proteomic method identifies proteins nitrated in vivo during inflammatory challenge. Proceedings of the National Academy of Sciences of the United States of America. 2001;98(21):12056–61. doi: 10.1073/pnas.221269198 11593016 10.1073/pnas.221269198 PMC59826

[69] Lopez A, Garcia JA, Escames G, Venegas C, Ortiz F, Lopez LC, et al Melatonin protects the mitochondria from oxidative damage reducing oxygen consumption, membrane potential, and superoxide anion production. JPineal Res. 2009;46(2):188–98. 19054298 10.1111/j.1600-079X.2008.00647.x

[70] Borah A, Mohanakumar KP. Melatonin inhibits 6-hydroxydopamine production in the brain to protect against experimental parkinsonism in rodents. Journal of pineal research. 2009;47(4):293–300. doi: 10.1111/j.1600-079X.2009.00713.x 19796048 10.1111/j.1600-079X.2009.00713.x

[71] Niranjan R, Nath C, Shukla R. The mechanism of action of MPTP-induced neuroinflammation and its modulation by melatonin in rat astrocytoma cells, C6. Free radical research. 2010;44(11):1304–16. doi: 10.3109/10715762.2010.501080 20815783 10.3109/10715762.2010.501080

[72] Hibaoui Y, Roulet E, Ruegg UT. Melatonin prevents oxidative stress-mediated mitochondrial permeability transition and death in skeletal muscle cells. Journal of pineal research. 2009;47(3):238–52. doi: 10.1111/j.1600-079X.2009.00707.x 19664004 10.1111/j.1600-079X.2009.00707.x

[73] Pyle A. Anugrha H. Kurzawa-Akanbi M. Yarnall A. Burn D.Hudson, et al Reduced mitochondrial DNA copy number is a biomarker of Parkinson's disease. Neurobiol Aging. 2016; 38: 216 e7–10. 10.1016/j.neurobiolaging.2015.10.033 PMC4759605 26639155

[74] Dolle C. Flones I. Nido G. S. Miletic H. Osuagwu N. Kristoffersen, et al Defective mitochondrial DNA homeostasis in the substantia nigra in Parkinson disease. Nat Commun. 2016; 7: 13548 doi: 10.1038/ncomms13548 27874000 10.1038/ncomms13548 PMC5121427

[75] Tzoulis C. Tran G. T. Schwarzlmuller T. Specht K. Haugarvoll K. Balafkan N. et al Severe nigrostriatal degeneration without clinical parkinsonism in patients with polymerase gamma mutations. Brain. 2013; 136(8): 2393–404. 23625061 10.1093/brain/awt103

[76] Semak I, Naumova M, Korik E, Terekhovich V, Wortsman J, Slominski A. A novel metabolic pathway of melatonin: oxidation by cytochrome C. Biochemistry. 2005;44(26):9300–7. doi: 10.1021/bi050202d 15981996 10.1021/bi050202d

[77] Hardeland R, Poeggeler B. Melatonin and synthetic melatonergic agonists: actions and metabolism in the central nervous system. Central nervous system agents in medicinal chemistry. 2012;12(3):189–216. 22640220 10.2174/187152412802430129

[78] Hardeland R. Melatonin, hormone of darkness and more: occurrence, control mechanisms, actions and bioactive metabolites. Cellular and molecular life sciences: CMLS. 2008;65(13):2001–18. doi: 10.1007/s00018-008-8001-x 18344019 10.1007/s00018-008-8001-x PMC11131831

[79] Patki G, Lau YS. Melatonin protects against neurobehavioral and mitochondrial deficits in a chronic mouse model of Parkinson's disease. Pharmacology, biochemistry, and behavior. 2011;99(4):704–11. doi: 10.1016/j.pbb.2011.06.026 21741988 10.1016/j.pbb.2011.06.026 PMC3156390

