# Supplementary material for: Mitochondrial impairment and melatonin protection in parkinsonian mice do not depend of inducible or neuronal nitric oxide synthases
Source: PLoS One. 2017 Aug 11;12(8):e0183090. doi: 10.1371/journal.pone.0183090 (PMC5553810; doi:10.1371/journal.pone.0183090)
Supplement: S1 File — (DOCX) [file pone.0183090.s002.docx]

**FIG. 1. MELATONIN LEVELS IN MITOCHONDRIA**

**iNOS^+/+^ mice**

| **VARIABLES** | | | | | |
| --- | --- | --- | --- | --- | --- |
|  |  | Mean | STDEV | SEM | N |
| **SN** | Untreated | 78.7 | 6.140032573 | 2.745906044 | 6 |
|  | MPTP | 85.6 | 6.580273551 | 2.942787794 | 6 |
|  | MPTP+aMT | 176.06 | 17.91976562 | 8.013962815 | 6 |
| **ST** | Untreated | 66.4 | 8.384509527 | 3.749666652 | 6 |
|  | MPTP | 53.8 | 5.540758071 | 2.477902339 | 6 |
|  | MPTP+aMT | 122 | 6.964194139 | 3.1144823 | 6 |

| **2 way ANOVA followed by Tukey's multiple comparisons test** | | | | | |
| --- | --- | --- | --- | --- | --- |
| *Groups of comparison* | *Mean Diff.* | *95.00% CI of diff.* | *Significant?* | *Summary* | *Adjusted P Value* |
| SN:Control vs. SN:MPTP | -6.8 | -25.49 to 11.89 | No | ns | 0.8662 |
| SN:Control vs. SN:MPTP+aMT | -97.2 | -115.9 to -78.51 | Yes | **** | <0.0001 |
| ST:Control vs. ST:MPTP | 12.6 | -6.089 to 31.29 | No | ns | 0.328 |
| ST:Control vs. ST:MPTP+aMT | -55.6 | -74.29 to -36.91 | Yes | **** | <0.0001 |
| SN:Control vs. ST:Control | 12.4 | -6.289 to 31.09 | No | ns | 0.3446 |
| SN:MPTP vs. ST:MPTP | 31.8 | 13.11 to 50.49 | Yes | *** | 0.0003 |
| SN:MPTP+aMT vs. ST:MPTP+aMT | 54 | 35.31 to 72.69 | Yes | **** | <0.0001 |
|  |  |  |  |  |  |

| **VARIABLES** | | | | | |
| --- | --- | --- | --- | --- | --- |
|  |  | Mean | STDEV | SEM | N |
| **SN** | Untreated | 100.5 | 12.68857754 | 5.674504384 | 6 |
|  | MPTP | 98.42 | 11.19741041 | 5.007634172 | 6 |
|  | MPTP+aMT | 193.06 | 17.91837046 | 8.01333888 | 6 |
| **ST** | Untreated | 77.6 | 3.049590136 | 1.36381817 | 6 |
|  | MPTP | 91.2 | 4.91934955 | 2.2 | 6 |
|  | MPTP+aMT | 107.94 | 9.196086124 | 4.11261474 | 6 |

**iNOS^-/-^ mice**

| **2 way ANOVA followed by Tukey's multiple comparisons test** | | | | | |
| --- | --- | --- | --- | --- | --- |
| *Groups of comparison* | *Mean Diff.* | *95.00% CI of diff.* | *Significant?* | *Summary* | *Adjusted P Value* |
| SN:Control vs. SN:MPTP | 2 | -19.36 to 23.36 | No | ns | 0.9997 |
| SN:Control vs. SN:MPTP+aMT | -92.6 | -114 to -71.24 | Yes | **** | <0.0001 |
| ST:Control vs. ST:MPTP | -13.6 | -34.96 to 7.759 | No | ns | 0.388 |
| ST:Control vs. ST:MPTP+aMT | -30.2 | -51.56 to -8.841 | Yes | ** | 0.0025 |
| SN:Control vs. ST:Control | 22.8 | 1.441 to 44.16 | Yes | * | 0.0316 |
| SN:MPTP vs. ST:MPTP | 7.2 | -14.16 to 28.56 | No | ns | 0.8987 |
| SN:MPTP+aMT vs. ST:MPTP+aMT | 85.2 | 63.84 to 106.6 | Yes | **** | <0.0001 |

**nNOS^+/+^ mice**

| **VARIABLES** | | | | | |
| --- | --- | --- | --- | --- | --- |
|  |  | Mean | STDEV | SEM | N |
| **SN** | Untreated | 88.62 | 10.50580792 | 4.698340132 | 6 |
|  | MPTP | 78.86 | 38.18897747 | 17.07862992 | 6 |
|  | MPTP+aMT | 175.4 | 16.05615147 | 7.180529228 | 6 |
| **ST** | Untreated | 70.24 | 4.38041094 | 1.958979326 | 6 |
|  | MPTP | 85.88 | 7.278186587 | 3.254903992 | 6 |
|  | MPTP+aMT | 115.5 | 4.301162634 | 1.923538406 | 6 |

|  | **2 way ANOVA followed by Tukey's multiple comparisons test** | | | | | |
| --- | --- | --- | --- | --- | --- | --- |
|  | *Groups of comparison* | *Mean Diff.* | *95.00% CI of diff.* | *Significant?* | *Summary* | *Adjusted P Value* |
|  | SN:Control vs. SN:MPTP | 9.8 | -25.13 to 44.73 | No | ns | 0.9506 |
|  | SN:Control vs. SN:MPTP+aMT | -86.8 | -121.7 to -51.87 | Yes | **** | <0.0001 |
|  | ST:Control vs. ST:MPTP | -15.6 | -50.53 to 19.33 | No | ns | 0.7375 |
|  | ST:Control vs. ST:MPTP+aMT | -45.2 | -80.13 to -10.27 | Yes | ** | 0.0062 |
|  | SN:Control vs. ST:Control | 18.4 | -16.53 to 53.33 | No | ns | 0.5886 |
|  | SN:MPTP vs. ST:MPTP | -7 | -41.93 to 27.93 | No | ns | 0.9885 |
|  | SN:MPTP+aMT vs. ST:MPTP+aMT | 60 | 25.07 to 94.93 | Yes | *** | 0.0002 |
|  |  |  |  |  |  |  |

**nNOS^-/-^ mice**

| **VARIABLES** | | | | | |
| --- | --- | --- | --- | --- | --- |
|  |  | Mean | STDEV | SEM | N |
| **SN** | Untreated | 78.22 | 4.97413309 | 2.224499944 | 6 |
|  | MPTP | 86.24 | 12.12798417 | 5.423799406 | 6 |
|  | MPTP+aMT | 145.44 | 8.394522023 | 3.754144377 | 6 |
| **ST** | Untreated | 68.06 | 6.64213821 | 2.970454511 | 6 |
|  | MPTP | 85.06 | 5.537869626 | 2.476610587 | 6 |
|  | MPTP+aMT | 114.26 | 5.864128239 | 2.622517874 | 6 |

| **2 way ANOVA followed by Tukey's multiple comparisons test** | | | | | |
| --- | --- | --- | --- | --- | --- |
| *Groups of comparison* | *Mean Diff.* | *95.00% CI of diff.* | *Significant?* | *Summary* | *Adjusted P Value* |
| SN:Control vs. SN:MPTP | -8 | -22.92 to 6.923 | No | ns | 0.5709 |
| SN:Control vs. SN:MPTP+aMT | -67.2 | -82.12 to -52.28 | Yes | **** | <0.0001 |
| ST:Control vs. ST:MPTP | -17 | -31.92 to -2.077 | Yes | * | 0.0191 |
| ST:Control vs. ST:MPTP+aMT | -46.2 | -61.12 to -31.28 | Yes | **** | <0.0001 |
| SN:Control vs. ST:Control | 10.2 | -4.723 to 25.12 | No | ns | 0.314 |
| SN:MPTP vs. ST:MPTP | 1.2 | -13.72 to 16.12 | No | ns | 0.9999 |
| SN:MPTP+aMT vs. ST:MPTP+aMT | 31.2 | 16.28 to 46.12 | Yes | **** | <0.0001 |

**FIG. 2. NOS ACTIVITY**

**SN INOS MICE**

| **VARIABLES** | | | | | | | |
| --- | --- | --- | --- | --- | --- | --- | --- |
|  |  | ***iNOS activity*** | | | ***nNOS activity*** | | |
|  |  | Mean | STDEV | N | Mean | STDEV | N |
| **iNOS+/+** | Untreated | 19.53 | 2.1 | 6 | 13.63 | 1.05 | 6 |
|  | MPTP | 60.93 | 4.89 | 7 | 19.01 | 4.03 | 6 |
|  | MPTP+aMT | 18.32 | 5.3 | 6 | 12.58 | 1.87 | 7 |
| **iNOS-/-** | Untreated | 0 | 0 | 6 | 15.74 | 4.84 | 6 |
|  | MPTP | 0 | 0 | 7 | 20.13 | 2.14 | 6 |
|  | MPTP+aMT | 0 | 0 | 6 | 12.49 | 5.01 | 6 |

| *Groups of comparison* | *Mean Diff.* | *95.00% CI of diff.* | *Significant?* | *Summary* | *Adjusted P Value* |
| --- | --- | --- | --- | --- | --- |
| iNOS actv (iNOS+/+):control vs. iNOS actv (iNOS+/+):MPTP | -41.4 | -47.87 to -34.93 | Yes | **** | <0.0001 |
| iNOS actv (iNOS+/+):control vs. iNOS actv (iNOS+/+):MPTP+aMT | 1.21 | -5.26 to 7.68 | No | ns | >0.9999 |
| iNOS actv (iNOS+/+):MPTP vs. iNOS actv (iNOS+/+):MPTP+aMT | 42.61 | 36.14 to 49.08 | Yes | **** | <0.0001 |
| nNOS actv (iNOS+/+):control vs. nNOS actv (iNOS+/+):MPTP | -5.38 | -11.85 to 1.09 | No | ns | 0.1952 |
| nNOS actv (iNOS+/+):control vs. nNOS actv (iNOS+/+):MPTP+aMT | 1.05 | -5.42 to 7.52 | No | ns | >0.9999 |
| nNOS actv (iNOS+/+):MPTP vs. nNOS actv (iNOS+/+):MPTP+aMT | 6.43 | -0.04008 to 12.9 | No | ns | 0.0529 |
| iNOS actv (iNOS-/-):control vs. iNOS actv (iNOS-/-):MPTP | 0 | -6.47 to 6.47 | No | ns | >0.9999 |
| iNOS actv (iNOS-/-):control vs. iNOS actv (iNOS-/-):MPTP+aMT | 0 | -6.47 to 6.47 | No | ns | >0.9999 |
| iNOS actv (iNOS-/-):MPTP vs. iNOS actv (iNOS-/-):MPTP+aMT | 0 | -6.47 to 6.47 | No | ns | >0.9999 |

**FIG. 2. NOS ACTIVITY**

**SN nNOS MICE**

| **VARIABLES** | | | | | | | |
| --- | --- | --- | --- | --- | --- | --- | --- |
|  |  | ***iNOS activity*** | | | ***nNOS activity*** | | |
|  |  | Mean | STDEV | N | Mean | STDEV | N |
| **nNOS+/+** | Untreated | 20.15 | 2.5 | 6 | 11.25 | 1.61 | 6 |
|  | MPTP | 65.32 | 5.02 | 6 | 17.45 | 3.78 | 6 |
|  | MPTP+aMT | 18.32 | 5.3 | 6 | 14.97 | 3.34 | 6 |
| **nNOS-/-** | Untreated | 24.12 | 2.47 | 7 | 0 | 0 | 6 |
|  | MPTP | 70.6 | 4.63 | 6 | 0 | 0 | 6 |
|  | MPTP+aMT | 26.72 | 2.15 | 6 | 0 | 0 | 7 |

| \| **2 way ANOVA followed by Tukey's multiple comparisons test** \| \| \| \| \| \| \| --- \| --- \| --- \| --- \| --- \| --- \| \| *Groups of comparison* \| *Mean Diff.* \| *95.00% CI of diff.* \| *Significant?* \| *Summary* \| *Adjusted P Value* \| \| iNOS actv (nNOS+/+):control vs. iNOS actv (nNOS+/+):MPTP \| -45.17 \| -51.14 to -39.2 \| Yes \| **** \| <0.0001 \| \| iNOS actv (nNOS+/+):control vs. iNOS actv (nNOS+/+):MPTP+aMT \| 1.83 \| -4.142 to 7.802 \| No \| ns \| 0.9959 \| \| iNOS actv (nNOS+/+):MPTP vs. iNOS actv (nNOS+/+):MPTP+aMT \| 47 \| 41.03 to 52.97 \| Yes \| **** \| <0.0001 \| \| nNOS actv (nNOS+/+):control vs. nNOS actv (nNOS+/+):MPTP \| -6.2 \| -12.4 to 0.0009053 \| No \| ns \| 0.0501 \| \| nNOS actv (nNOS+/+):control vs. nNOS actv (nNOS+/+):MPTP+aMT \| -4.72 \| -10.69 to 1.252 \| No \| ns \| 0.2578 \| \| nNOS actv (nNOS+/+):MPTP vs. nNOS actv (nNOS+/+):MPTP+aMT \| 3.58 \| -2.392 to 9.552 \| No \| ns \| 0.6663 \| \| iNOS actv (nNOS-/-):control vs. iNOS actv (nNOS-/-):MPTP \| -46.48 \| -52.45 to -40.51 \| Yes \| **** \| <0.0001 \| \| iNOS actv (nNOS-/-):control vs. iNOS actv (nNOS-/-):MPTP+aMT \| -2.6 \| -8.572 to 3.372 \| No \| ns \| 0.9402 \| \| iNOS actv (nNOS-/-):MPTP vs. iNOS actv (nNOS-/-):MPTP+aMT \| 43.88 \| 37.91 to 49.85 \| Yes \| **** \| <0.0001 \| | | | |
| --- | --- | --- | --- | --- | --- | --- | --- | --- | --- | --- | --- | --- | --- | --- | --- | --- | --- | --- | --- | --- | --- | --- | --- | --- | --- | --- | --- | --- | --- | --- | --- | --- | --- | --- | --- | --- | --- | --- | --- | --- | --- | --- | --- | --- | --- | --- | --- | --- | --- | --- | --- | --- | --- | --- | --- | --- | --- | --- | --- | --- | --- | --- | --- | --- | --- | --- | --- | --- | --- |
|  | | | |
|  |  |  |  |

**FIG. 2. NOS ACTIVITY**

**ST INOS MICE**

| **VARIABLES** | | | | | | | |
| --- | --- | --- | --- | --- | --- | --- | --- |
|  |  | ***iNOS activity*** | | | ***nNOS activity*** | | |
|  |  | Mean | STDEV | N | Mean | STDEV | N |
| **iNOS+/+** | Untreated | 25.21 | 2.8 | 6 | 27.62 | 4.89 | 6 |
|  | MPTP | 52.18 | 5.01 | 6 | 25.38 | 4.33 | 6 |
|  | MPTP+aMT | 28.54 | 2.63 | 6 | 24.97 | 3.66 | 7 |
| **iNOS-/-** | Untreated | 0 | 0 | 7 | 25.04 | 3.14 | 6 |
|  | MPTP | 0 | 0 | 6 | 26.4 | 3.53 | 6 |
|  | MPTP+aMT | 0 | 0 | 6 | 25.42 | 2.87 | 6 |

|  |
| --- |

| \| **2 way ANOVA followed by Tukey's multiple comparisons test** \| \| \| \| \| \| \| --- \| --- \| --- \| --- \| --- \| --- \| \| *Groups of comparison* \| *Mean Diff.* \| *95.00% CI of diff.* \| *Significant?* \| *Summary* \| *Adjusted P Value* \| \| iNOS actv (iNOS+/+):control vs. iNOS actv (iNOS+/+):MPTP \| -27.3 \| -33.65 to -20.95 \| Yes \| **** \| <0.0001 \| \| iNOS actv (iNOS+/+):control vs. iNOS actv (iNOS+/+):MPTP+aMT \| -2.22 \| -8.567 to 4.127 \| No \| ns \| 0.9879 \| \| iNOS actv (iNOS+/+):MPTP vs. iNOS actv (iNOS+/+):MPTP+aMT \| 25.08 \| 18.73 to 31.43 \| Yes \| **** \| <0.0001 \| \| nNOS actv (iNOS+/+):control vs. nNOS actv (iNOS+/+):MPTP \| 2.24 \| -4.107 to 8.587 \| No \| ns \| 0.987 \| \| nNOS actv (iNOS+/+):control vs. nNOS actv (iNOS+/+):MPTP+aMT \| 2.65 \| -3.697 to 8.997 \| No \| ns \| 0.9549 \| \| nNOS actv (iNOS+/+):MPTP vs. nNOS actv (iNOS+/+):MPTP+aMT \| 0.41 \| -5.937 to 6.757 \| No \| ns \| >0.9999 \| \| nNOS actv (iNOS-/-):control vs. nNOS actv (iNOS-/-):MPTP \| -1.36 \| -7.707 to 4.987 \| No \| ns \| 0.9998 \| \| nNOS actv (iNOS-/-):control vs. nNOS actv (iNOS-/-):MPTP+aMT \| -0.38 \| -6.727 to 5.967 \| No \| ns \| >0.9999 \| \| nNOS actv (iNOS-/-):MPTP vs. nNOS actv (iNOS-/-):MPTP+aMT \| 0.98 \| -5.367 to 7.327 \| No \| ns \| >0.9999 \| |
| --- | --- | --- | --- | --- | --- | --- | --- | --- | --- | --- | --- | --- | --- | --- | --- | --- | --- | --- | --- | --- | --- | --- | --- | --- | --- | --- | --- | --- | --- | --- | --- | --- | --- | --- | --- | --- | --- | --- | --- | --- | --- | --- | --- | --- | --- | --- | --- | --- | --- | --- | --- | --- | --- | --- | --- | --- | --- | --- | --- | --- | --- | --- | --- | --- | --- | --- |
|  |

**FIG. 2. NOS ACTIVITY**

**ST nNOS MICE**

| **VARIABLES** | | | | | | | |
| --- | --- | --- | --- | --- | --- | --- | --- |
|  |  | ***iNOS activity*** | | | ***nNOS activity*** | | |
|  |  | Mean | STDEV | N | Mean | STDEV | N |
| **nNOS+/+** | Untreated | 24.54 | 3.02 | 6 | 22.82 | 3.78 | 6 |
|  | MPTP | 49.59 | 4.55 | 6 | 24.31 | 4.38 | 6 |
|  | MPTP+aMT | 26.58 | 2.17 | 7 | 23.35 | 3.36 | 6 |
| **nNOS-/-** | Untreated | 20.99 | 1.87 | 6 | 0 | 0 | 7 |
|  | MPTP | 52.01 | 5.12 | 6 | 0 | 0 | 6 |

| **2 way ANOVA followed by Tukey's multiple comparisons test** | | | | | |
| --- | --- | --- | --- | --- | --- |
| *Groups of comparison* | *Mean Diff.* | *95.00% CI of diff.* | *Significant?* | *Summary* | *Adjusted P Value* |
| iNOS actv (nNOS+/+):control vs. iNOS actv (nNOS+/+):MPTP | -25.05 | -31.06 to -19.04 | Yes | **** | <0.0001 |
| iNOS actv (nNOS+/+):control vs. iNOS actv (nNOS+/+):MPTP+aMT | -2.04 | -8.051 to 3.971 | No | ns | 0.9905 |
| iNOS actv (nNOS+/+):MPTP vs. iNOS actv (nNOS+/+):MPTP+aMT | 23.01 | 17 to 29.02 | Yes | **** | <0.0001 |
| nNOS actv (nNOS+/+):control vs. nNOS actv (nNOS+/+):MPTP | -1.49 | -7.501 to 4.521 | No | ns | 0.9994 |
| nNOS actv (nNOS+/+):control vs. nNOS actv (nNOS+/+):MPTP+aMT | -0.53 | -6.541 to 5.481 | No | ns | >0.9999 |
| nNOS actv (nNOS+/+):MPTP vs. nNOS actv (nNOS+/+):MPTP+aMT | 0.96 | -5.051 to 6.971 | No | ns | >0.9999 |
| iNOS actv (nNOS-/-):control vs. iNOS actv (nNOS-/-):MPTP | -31.02 | -37.03 to -25.01 | Yes | **** | <0.0001 |
| iNOS actv (nNOS-/-):control vs. iNOS actv (nNOS-/-):MPTP+aMT | -2.15 | -8.161 to 3.861 | No | ns | 0.9856 |
| iNOS actv (nNOS-/-):MPTP vs. iNOS actv (nNOS-/-):MPTP+aMT | 28.87 | 22.86 to 34.88 | Yes | **** | <0.0001 |

**FIG. 3. COMPLEX I ACTIVITY**

| **iNOS^+/+^ mice** | | | | | | |
| --- | --- | --- | --- | --- | --- | --- |
| **VARIABLES** | | | | | | |
|  |  | Mean | STDEV | SEM | N |  |
| **SN** | Untreated | 9.192 | 1.079 | 0.4405 | 6 |  |
|  | MPTP | 2.724 | 0.6178 | 0.233506 | 7 |  |
|  | MPTP+aMT | 8.618 | 0.7908 | 0.322843 | 6 |  |
| **ST** | Untreated | 7.154 | 0.1006 | 0.04107 | 6 |  |
|  | MPTP | 5.21 | 0.3104 | 0.109743 | 8 |  |
|  | MPTP+aMT | 8.066 | 1.292 | 0.527457 | 6 |  |
|  | | | | | | |
| **2 way ANOVA followed by Tukey's multiple comparisons test** | | | | | | |
|  | *Groups of comparison* | *Mean Diff.* | *95.00% CI of diff.* | *Significant?* | *Summary* | *Adjusted P Value* |
|  | SN:control vs. SN:MPTP | 6.468 | 5.044 to 7.892 | Yes | **** | <0.0001 |
|  | SN:control vs. SN:MPTP+aMT | 0.574 | -0.8503 to 1.998 | No | ns | 0.8209 |
|  | SN:MPTP vs. SN:MPTP+aMT | -5.894 | -7.318 to -4.47 | Yes | **** | <0.0001 |
|  | ST:control vs. ST:MPTP | 1.944 | 0.5197 to 3.368 | Yes | ** | 0.0031 |
|  | ST:control vs. ST:MPTP+aMT | -0.912 | -2.336 to 0.5123 | No | ns | 0.3947 |
|  | ST:MPTP vs. ST:MPTP+aMT | -2.856 | -4.28 to -1.432 | Yes | **** | <0.0001 |
|  | SN:control vs. ST:control | 2.038 | 0.6137 to 3.462 | Yes | ** | 0.0018 |

| **iNOS^-/-^ mice** | | | | | | |
| --- | --- | --- | --- | --- | --- | --- |
| **VARIABLES** | | | | | | |
|  |  | Mean | STDEV | SEM | N |  |
| **SN** | Untreated | 8.683 | 0.5981 | 0.244173 | 6 |  |
|  | MPTP | 2.312 | 0.8319 | 0.314429 | 7 |  |
|  | MPTP+aMT | 6.087 | 0.3398 | 0.138723 | 6 |  |
| **ST** | Untreated | 6.549 | 0.6164 | 0.251644 | 6 |  |
|  | MPTP | 3.721 | 0.3938 | 0.160768 | 6 |  |
|  | MPTP+aMT | 6.211 | 0.9022 | 0.368322 | 6 |  |
|  | | | | | | |
| **2 way ANOVA followed by Tukey's multiple comparisons test** | | | | | | |
|  | *Groups of comparison* | *Mean Diff.* | *95.00% CI of diff.* | *Significant?* | *Summary* | *Adjusted P Value* |
|  | SN:control vs. ST:MPTP | 4.962 | 3.825 to 6.099 | Yes | **** | <0.0001 |
|  | SN:control vs. ST:MPTP+aMT | 2.472 | 1.335 to 3.609 | Yes | **** | <0.0001 |
|  | SN:MPTP vs. SN:MPTP+aMT | -3.775 | -4.912 to -2.638 | Yes | **** | <0.0001 |
|  | ST:control vs. ST:MPTP | 2.828 | 1.691 to 3.965 | Yes | **** | <0.0001 |
|  | ST:control vs. ST:MPTP+aMT | 0.338 | -0.7988 to 1.475 | No | ns | 0.9424 |
|  | ST:MPTP vs. ST:MPTP+aMT | -2.49 | -3.627 to -1.353 | Yes | **** | <0.0001 |
|  | SN:control vs. ST:control | 2.134 | 0.9972 to 3.271 | Yes | **** | <0.0001 |

**FIG. 3. COMPLEX I ACTIVITY**

| **nNOS^+/+^ mice** | | | | | | |
| --- | --- | --- | --- | --- | --- | --- |
| **VARIABLES** | | | | | | |
|  |  | Mean | STDEV | SEM | N |  |
| **SN** | Untreated | 11.5 | 0.8534 | 0.348399 | 6 |  |
|  | MPTP | 4.768 | 0.7136 | 0.269715 | 7 |  |
|  | MPTP+aMT | 9.655 | 0.4237 | 0.172975 | 6 |  |
| **ST** | Untreated | 10.22 | 1.611 | 0.657688 | 6 |  |
|  | MPTP | 5.573 | 0.6737 | 0.275037 | 6 |  |
|  | MPTP+aMT | 8.9 | 0.6662 | 0.271975 | 6 |  |
|  | | | | | | |
| **2 way ANOVA followed by Tukey's multiple comparisons test** | | | | | | |
|  | *Groups of comparison* | *Mean Diff.* | *95.00% CI of diff.* | *Significant?* | *Summary* | *Adjusted P Value* |
|  | SN:control vs. SN:MPTP | 6.732 | 5.143 to 8.321 | Yes | **** | <0.0001 |
|  | SN:control vs. SN:MPTP+aMT | 1.845 | 0.2564 to 3.434 | Yes | * | 0.0155 |
|  | SN:MPTP vs. SN:MPTP+aMT | -4.887 | -6.476 to -3.298 | Yes | **** | <0.0001 |
|  | ST:control vs. ST:MPTP | 4.647 | 3.058 to 6.236 | Yes | **** | <0.0001 |
|  | ST:control vs. ST:MPTP+aMT | 1.32 | -0.2686 to 2.909 | No | ns | 0.1481 |
|  | ST:MPTP vs. ST:MPTP+aMT | -3.327 | -4.916 to -1.738 | Yes | **** | <0.0001 |
|  | SN:control vs. ST:control | 1.28 | -0.3086 to 2.869 | No | ns | 0.1714 |

| **nNOS^-/-^ mice** | | | | | | |
| --- | --- | --- | --- | --- | --- | --- |
| **VARIABLES** | | | | | | |
|  |  | Mean | STDEV | SEM | N |  |
| **SN** | Untreated | 5.13 | 1.153249808 | 0.470812 | 6 |  |
|  | MPTP | 1.98 | 0.627122247 | 0.256022 | 6 |  |
|  | MPTP+aMT | 3.3 | 0.063 | 0.02572 | 6 |  |
| **ST** | Untreated | 10.43 | 1.976213447 | 0.806786 | 6 |  |
|  | MPTP | 3.93 | 0.91163294 | 0.372173 | 6 |  |
|  | MPTP+aMT | 7.25 | 2.004583595 | 0.818368 | 6 |  |
|  | | | | | | |
| **2 way ANOVA followed by Tukey's multiple comparisons test** | | | | | | |
|  | *Groups of comparison* | *Mean Diff.* | *95.00% CI of diff.* | *Significant?* | *Summary* | *Adjusted P Value* |
|  | SN:control vs. SN:MPTP | 3.15 | 0.8289 to 5.471 | Yes | ** | 0.0033 |
|  | SN:control vs. SN:MPTP+aMT | 1.83 | -0.4911 to 4.151 | No | ns | 0.1891 |
|  | SN:MPTP vs. SN:MPTP+aMT | -1.32 | -3.641 to 1.001 | No | ns | 0.5239 |
|  | ST:control vs. ST:MPTP | 6.5 | 4.179 to 8.821 | Yes | **** | <0.0001 |
|  | ST:control vs. ST:MPTP+aMT | 3.18 | 0.8589 to 5.501 | Yes | ** | 0.003 |
|  | ST:MPTP vs. ST:MPTP+aMT | -3.32 | -5.641 to -0.9989 | Yes | ** | 0.0018 |

**FIG. 5. STATE 3 AND STATE 4 DURING RESPIRATION**

| **VARIABLES: O_2_FLUX (PMOL/S·MG PROT)** | | | | | | | | | |
| --- | --- | --- | --- | --- | --- | --- | --- | --- | --- |
|  |  |  |  | **C I+II** | | | **C II** | | |
|  |  |  |  | **Mean** | **STDEV** | **N** | **Mean** | **STDEV** | **N** |
| **Substatia nigra** | **iNOS^+/+^** | **State 3** | **control** | 1865.20 | 169.39 | 4 | 580.91 | 86.96 | 4 |
|  |  |  | **MPTP** | 534.75 | 157.29 | 4 | 428.10 | 74.86 | 4 |
|  |  |  | **MPTP+aMT** | 1300.76 | 161.25 | 5 | 622.45 | 78.82 | 5 |
|  |  | **State 4** | **control** | 785.82 | 49.03 | 4 | 550.05 | 83.82 | 4 |
|  |  |  | **MPTP** | 514.78 | 36.93 | 4 | 412.56 | 71.72 | 4 |
|  |  |  | **MPTP+aMT** | 651.32 | 40.89 | 5 | 578.42 | 75.68 | 5 |
|  | **iNOS^-/-^** | **State 3** | **control** | 1878.42 | 62.58 | 4 | 945.33 | 183.41 | 4 |
|  |  |  | **MPTP** | 1022.51 | 33.80 | 5 | 752.41 | 55.99 | 5 |
|  |  |  | **MPTP+aMT** | 1546.35 | 43.82 | 4 | 934.58 | 66.01 | 4 |
|  |  | **State 4** | **control** | 1006.43 | 164.57 | 4 | 822.86 | 135.37 | 4 |
|  |  |  | **MPTP** | 731.86 | 58.49 | 5 | 667.25 | 49.72 | 5 |
|  |  |  | **MPTP+aMT** | 1002.05 | 38.51 | 4 | 822.46 | 59.74 | 4 |
|  | **nNOS^+/+^** | **State 3** | **control** | 1552.29 | 142.49 | 5 | 705.44 | 178.60 | 5 |
|  |  |  | **MPTP** | 720.63 | 135.03 | 5 | 497.21 | 21.28 | 5 |
|  |  |  | **MPTP+aMT** | 1354.27 | 114.20 | 4 | 706.24 | 45.18 | 4 |
|  |  | **State 4** | **control** | 835.29 | 191.96 | 5 | 487.20 | 119.42 | 5 |
|  |  |  | **MPTP** | 656.62 | 189.30 | 5 | 449.96 | 17.12 | 5 |
|  |  |  | **MPTP+aMT** | 862.28 | 168.47 | 4 | 599.75 | 38.91 | 4 |
|  | **nNOS^-/-^** | **State 3** | **control** | 1374.00 | 126.34 | 4 | 540.64 | 39.94 | 4 |
|  |  |  | **MPTP** | 757.13 | 59.01 | 5 | 489.36 | 30.02 | 5 |
|  |  |  | **MPTP+aMT** | 1340.85 | 49.25 | 4 | 592.50 | 20.26 | 4 |
|  |  | **State 4** | **control** | 869.23 | 70.96 | 4 | 519.66 | 33.81 | 4 |
|  |  |  | **MPTP** | 582.92 | 43.92 | 5 | 479.16 | 34.17 | 5 |
|  |  |  | **MPTP+aMT** | 839.95 | 34.16 | 4 | 563.10 | 24.41 | 4 |

| **Striatum** | **iNOS^+/+^** | **State 3** | **control** | 1953.78 | 112.17 | 4 | 686.52 | 18.34 | 4 |
| --- | --- | --- | --- | --- | --- | --- | --- | --- | --- |
|  |  |  | **MPTP** | 1318.91 | 107.44 | 4 | 642.13 | 17.86 | 4 |
|  |  |  | **MPTP+aMT** | 1862.85 | 102.63 | 4 | 696.48 | 17.38 | 4 |
|  |  | **State 4** | **control** | 1083.97 | 78.40 | 4 | 631.58 | 17.58 | 4 |
|  |  |  | **MPTP** | 902.50 | 73.67 | 4 | 623.75 | 17.10 | 4 |
|  |  |  | **MPTP+aMT** | 1018.89 | 68.86 | 4 | 625.25 | 16.62 | 4 |
|  | **iNOS^-/-^** | **State 3** | **control** | 1642.75 | 122.89 | 4 | 825.13 | 101.12 | 4 |
|  |  |  | **MPTP** | 1259.90 | 112.50 | 5 | 724.94 | 15.45 | 5 |
|  |  |  | **MPTP+aMT** | 1511.93 | 111.49 | 4 | 754.20 | 89.72 | 4 |
|  |  | **State 4** | **control** | 872.91 | 42.28 | 4 | 602.30 | 112.05 | 4 |
|  |  |  | **MPTP** | 899.12 | 24.55 | 5 | 593.64 | 5.85 | 5 |
|  |  |  | **MPTP+aMT** | 807.55 | 30.88 | 4 | 580.65 | 100.65 | 4 |
|  | **nNOS^+/+^** | **State 3** | **control** | 1393.32 | 122.19 | 5 | 766.10 | 32.21 | 5 |
|  |  |  | **MPTP** | 719.95 | 62.24 | 5 | 651.87 | 49.40 | 5 |
|  |  |  | **MPTP+aMT** | 1344.38 | 122.19 | 4 | 745.65 | 32.21 | 4 |
|  |  | **State 4** | **control** | 805.71 | 101.59 | 5 | 565.26 | 72.72 | 5 |
|  |  |  | **MPTP** | 703.13 | 45.01 | 5 | 564.17 | 52.29 | 5 |
|  |  |  | **MPTP+aMT** | 790.87 | 82.60 | 4 | 584.72 | 72.72 | 4 |
|  | **nNOS^-/-^** | **State 3** | **control** | 1689.32 | 227.09 | 4 | 715.98 | 66.04 | 4 |
|  |  |  | **MPTP** | 1139.95 | 48.21 | 5 | 580.57 | 35.92 | 5 |
|  |  |  | **MPTP+aMT** | 1593.85 | 227.09 | 4 | 754.20 | 66.04 | 4 |
|  |  | **State 4** | **control** | 943.55 | 82.60 | 4 | 636.60 | 106.06 | 4 |
|  |  |  | **MPTP** | 718.15 | 46.36 | 5 | 523.43 | 65.14 | 5 |
|  |  |  | **MPTP+aMT** | 931.97 | 82.60 | 4 | 647.85 | 106.06 | 4 |

**STATISTICS**

**FIG. 6. mtDNA/nDNA ratio**

**VARIABLES**

**FIG. 7. mtDNA/nDNA ratio**

**VARIABLES**
